# Site-1 protease regulates skeletal stem cell population and osteogenic differentiation in mice

**DOI:** 10.1242/bio.032094

**Published:** 2018-02-01

**Authors:** Debabrata Patra, Elizabeth DeLassus, Jennifer Mueller, Grazia Abou-Ezzi, Linda J. Sandell

**Affiliations:** 1 Department of Orthopaedic Surgery, Washington University School of Medicine, St. Louis, MO 63110, USA; 2 Department of Biochemistry, Washington University School of Medicine, St. Louis, MO 63110, USA; 3 Department of Medicine, Oncology Division, Washington University School of Medicine, St. Louis, MO 63110, USA; 4 Department of Cell Biology and Physiology, Washington University School of Medicine, St. Louis, MO 63110, USA

**Keywords:** Site-1 protease, Skeletal stem cells, Osteopenia, Osterix

## Abstract

Site-1 protease (S1P) is a proprotein convertase with essential functions in the conversion of precursor proteins to their active form. In earlier studies, we demonstrated that S1P ablation in the chondrocyte lineage results in a drastic reduction in endochondral bone formation. To investigate the mechanistic contribution of S1P to bone development we ablated S1P in the osterix lineage in mice. S1P ablation in this lineage results in osteochondrodysplasia and variable degrees of early postnatal scoliosis. Embryonically, even though Runx2 and osterix expression are normal, S1P ablation results in a delay in vascular invasion and endochondral bone development. Mice appear normal when born, but by day 7 display pronounced dwarfism with fragile bones that exhibit significantly reduced mineral density, mineral apposition rate, bone formation rate and reduced osteoblasts indicating severe osteopenia. Mice suffer from a drastic reduction in bone marrow mesenchymal progenitors as analyzed by colony-forming unit-fibroblast assay. Fluorescence-activated cell sorting analysis of the skeletal mesenchyme harvested from bone marrow and collagenase-digested bone show a drastic reduction in hematopoietic lineage-negative, endothelial-negative, CD105*^+^* skeletal stem cells. Bone marrow mesenchymal progenitors are unable to differentiate into osteoblasts *in vitro*, with no effect on adipogenic differentiation. Postnatal mice have smaller growth plates with reduced hypertrophic zone. Thus, S1P controls bone development directly by regulating the skeletal progenitor population and their differentiation into osteoblasts.

This article has an associated First Person interview with the first author of the paper.

## Abstract (precis)

**Summary:** S1P governs a fundamental aspect of skeletal development and homeostasis, mainly the maintenance and osteogenic differentiation of skeletogenic stem cells that are a source of osteoblast and chondrocyte lineages.

## INTRODUCTION

Site-1 protease (S1P), coded by the *Mbtps1* gene (*membrane bound transcription factor protease, site 1*), is a proprotein convertase with vital roles in lipid homeostasis and the unfolded protein response (UPR) (Brown et al., 2000; Eberlé et al., 2004). These pathways are fundamental to cellular homeostasis and involve the activation of latent, endoplasmic reticulum (ER)-membrane-bound transcription factors. Of late, newer roles have been attributed to S1P and the transcription factors that it processes. OASIS (old astrocyte specifically induced substance), a S1P substrate, is necessary for *type I collagen* (*Col1a1* and *Col1a2*) expression in mice, critical for bone formation (Murakami et al., 2009). The processing of *N*-acetylglucosamine-1-phosphotransferase α/β-subunit precursor by S1P is essential to lysosome biogenesis, which impacts skeletal development (Marschner et al., 2011). In the zebrafish *gonzo* phenotype, S1P ablation results in both cartilage and lipid phenotypes, but ablation of SCAP [the SREBP (sterol responsive element binding protein) cleavage activating protein] results only in lipid phenotypes (Schlombs et al., 2003). This indicates that not all S1P functions are lipid regulated or mediated through transcription factors, and is indicative of additional roles for S1P that impact skeletal development.

We have demonstrated previously that S1P is essential to skeletal development. S1P ablation in osteochondroprogenitors in mice (S1P*^cko^*; via *Col2-Cre*) results in chondrodysplasia (Patra et al., 2014b, 2007). These mice show abnormal cartilage development with type IIB procollagen (pro-Col IIB) entrapment in the chondrocyte ER and a drastic reduction in type II collagen (Col II) protein in the cartilage. The pro-Col IIB entrapment is so drastic that it induces UPR and chondrocyte apoptosis; these mice also lack endochondral bone. Postnatal ablation of S1P in chondrocytes resulted in loss of hypertrophic chondrocyte (HC) differentiation, elimination of the primary growth plate and loss of bone growth (Patra et al., 2011). In these studies, the cartilage phenotype was deemed a primary consequence of the mutation and bone developmental defects a consequence of cartilage defect.

To investigate if S1P has a direct role in bone development, we ablated S1P in the osterix (Osx) lineage using *Osx-Cre* mice (Rodda and McMahon, 2006). Osx is a transcription factor expressed in late stages of endochondral ossification (Nishimura et al., 2012). It is expressed strongly in osteoblast precursors and required for osteoblast differentiation; it is also expressed in pre-HCs, where it is required for maturation. Our observation that postnatal S1P ablation obliterates HCs, coupled with the fact that HCs can transdifferentiate into osteoblasts (Park et al., 2015; Yang et al., 2014; Zhou et al., 2014), presented an opportunity to address the importance of S1P to bone development via its ablation in the Osx lineage. In this study, we show that S1P ablation in the Osx lineage drastically downregulates postnatal bone development resulting in osteopenia, indicating a direct role for S1P in bone development. Our mechanistic characterizations show that S1P is necessary to maintain the skeletal mesenchyme in the postnatal bone marrow. It is also required for the differentiation of mesenchymal progenitors into osteoblasts. Thus S1P is needed at multiple stages during bone development.

## RESULTS

### S1P ablation in the Osx lineage results in dwarfism

**Fig. 1.**
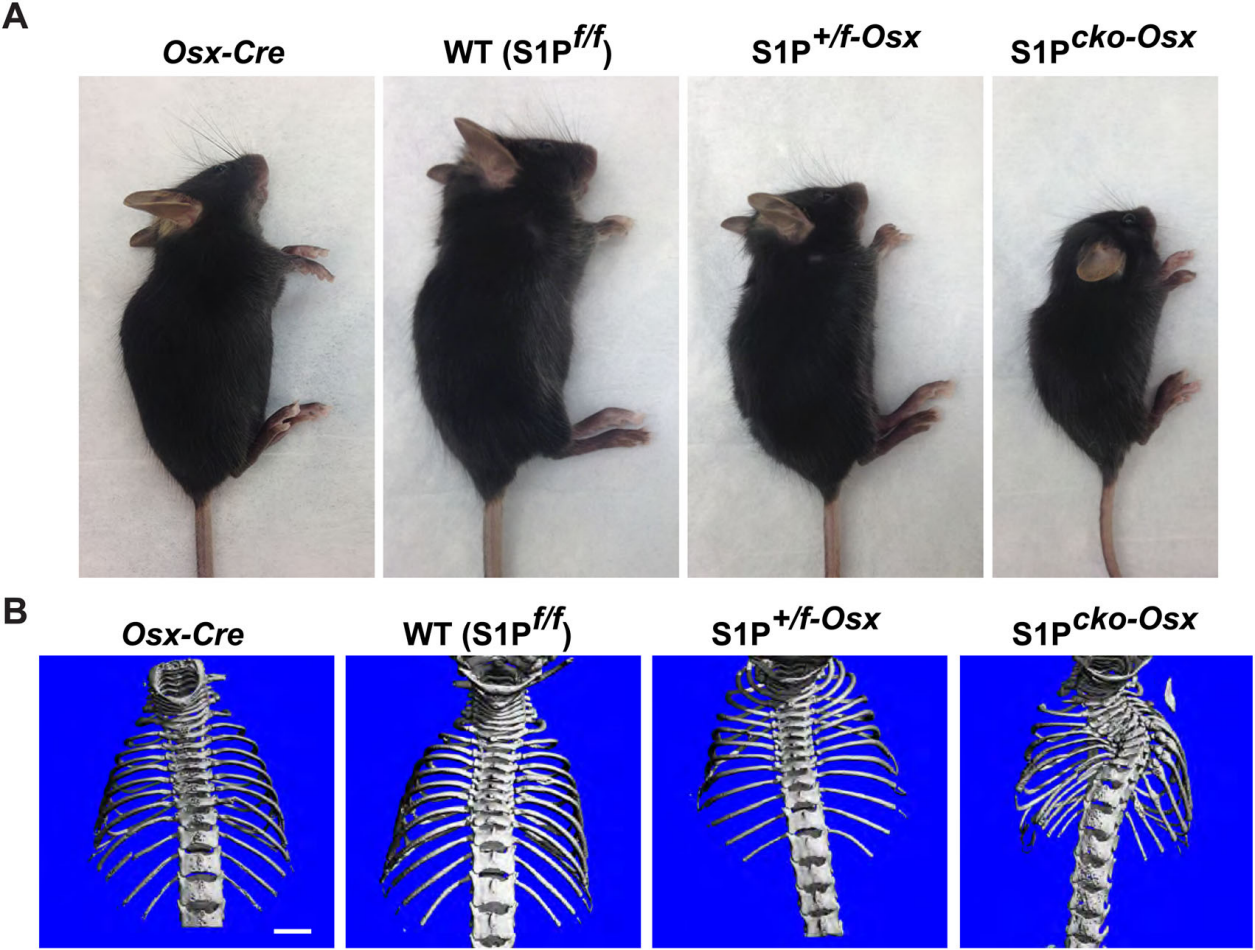
**Mice with S1P ablation in the Osx lineage.** (A) S1P ablation in the Osx lineage results in short-statured mice. Mice are 21 days postnatal (P21). (B) Images from μCT scans of P21 mice showing severe scoliosis in S1P*^cko^*^-Osx^. Scale bar: 2.5 mm.

To investigate roles for S1P in bone development, we used *Osx-Cre* mice to ablate S1P in the Osx lineage. Homozygous S1P-ablation in the Osx lineage (S1P*^cko^*^-*Osx*^ or Cko) results in dwarfism with very fragile bones that often break easily from normal cage activities. Heterozygously ablated S1P*^+/f^*^-*Osx*^ (Het) mice are intermediate in size to wild-type (WT) (S1P*^f/f^*) and S1P*^cko^*^-*Osx*^ mice and are smaller than *Osx-Cre* mice (Fig. 1A). S1P*^cko^*^-*Osx*^ mice often display varying degrees of scoliosis that at times is severe (Fig. 1B; Fig. S1) and is seen as early as 7-10 days postnatally (Fig. S1).

**Fig. 2.**
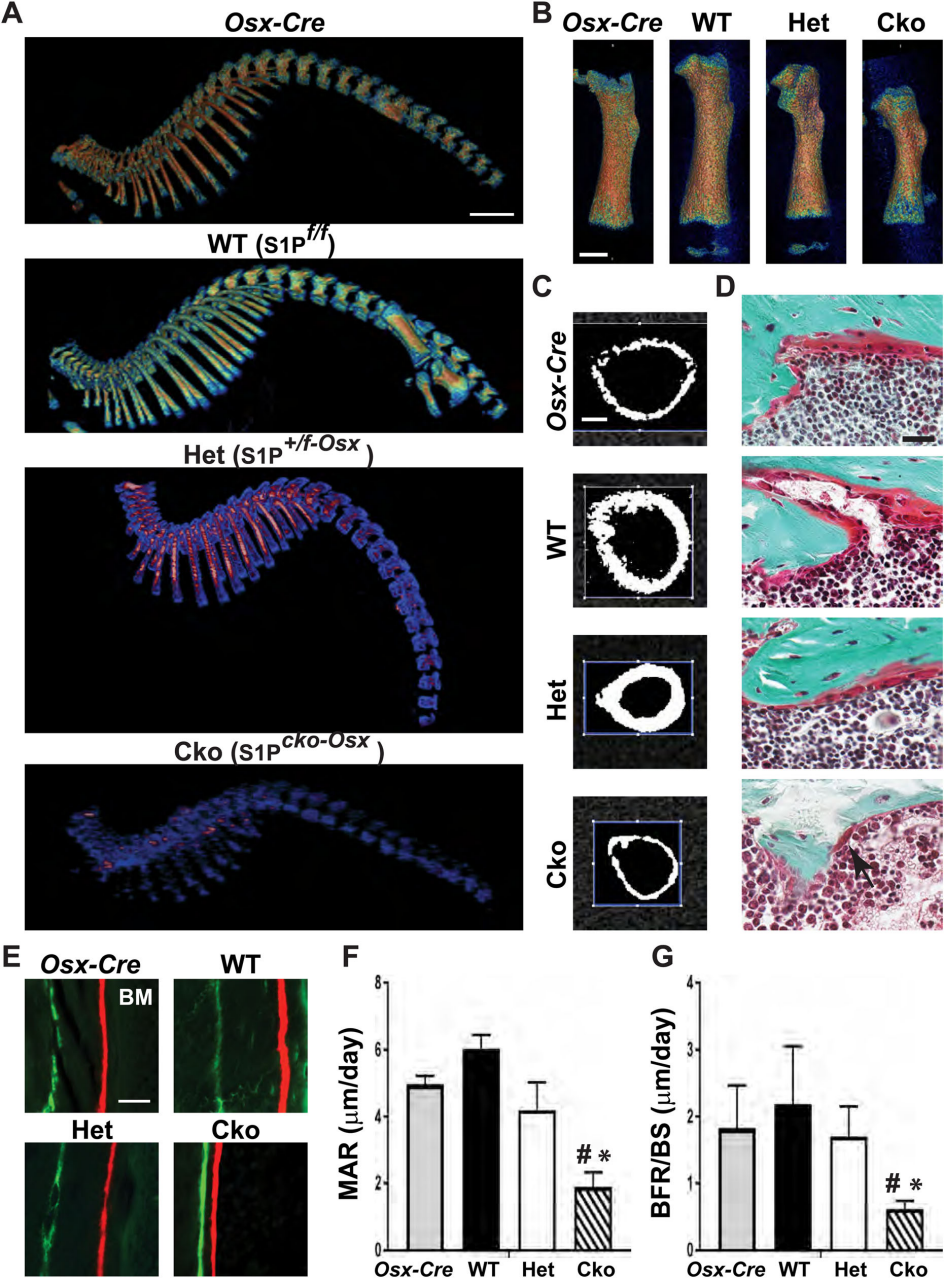
**Osteopenia due to S1P ablation in the Osx lineage.** (A,B) BMD heat maps generated for P7 *Osx-Cre*, WT (S1P*^f/f^*), S1P*^+/f^*^-Osx^ (Het) and S1P*^cko-Osx^* (Cko) spines (A) and femora (B) from μCT scans processed by OsiriX using Jet color scheme (window location, 1440; window width, 1890; for all images). A typical representation from several litters (*N*=3) is shown. (C) Mid-diaphyseal femoral cortical bone from μCT scans in P7 mice. (D) Gold-trichrome staining for osteoblasts (reddish) lining the endosteal in P28 cortical bone, drastically reduced in the Cko. Arrow in Cko points to a lone osteoblast. (E) Calcein (green)-alizarin (red) double-labeling of the endosteal surface of the femoral cortical bone in P28 mice (BM, bone marrow). (F) MAR (mean±s.d.; *N*=5). *P<0.0004 compared to WT/*Osx-Cre;* ^#^*P*=0.006 (compared to Het). (G) BFR/BS (mean±s.d.; *N*=5). *P=0.0004 (compared to WT); ^#^*P*=0.014 (compared to Het/*Osx-Cre*). Scale bars: (A) 2.5 mm; (B) 1 mm; (C) 0.25 mm; (D,E) 25 μm.

To investigate whether dwarfism is related to aberrant bone development we analyzed the skeletons of these mice by micro-computed tomography (μCT). Scanned images were developed by OsiriX software and Jet color scheme to generate bone mineral density (BMD) heat maps, in which yellowish-orange signifies high, and blue low, values for BMD. Fig. 2A and Fig. S2 show that bone development deviates very early and rapidly from normal, postnatally. When compared to postnatal day (P) 1 mice (Fig. S2A), P7 S1P*^cko-Osx^* (Cko) mice are severely osteopenic with drastically reduced BMD and smaller axial (Fig. 2A) and appendicular (Fig. 2B) skeletal elements; S1P*^+/f-Osx^* (Het) mice are intermediate to WT and S1P*^cko-Osx^* mice (Fig. 2A,B). While no noticeable differences in size and BMD are seen in P1 (Fig. S2A), reductions in size and BMD are visible at P5 in S1P-ablated mice in comparison to *Osx-Cre* or WT mice (Fig. S2B). In P7 Cko mice, the mid-diaphyseal cortical bone is smaller in width with thinner cortical bone when compared to WT mice (Fig. 2C); Het mice, though showing no decrease in cortical bone thickness, are smaller in width when compared to WT or *Osx-Cre* mice (Fig. 2C).

In dynamic histomorphometric analysis performed by calcein (green)-alizarin (red) double labeling of bone, Cko mice exhibited a significant reduction in both mineral apposition rate (MAR) (Fig. 2E,F) and bone formation rate/bone surface (BFR/BS) (Fig. 2G) that correlates well with the decrease in osteoblasts seen on the endosteal surface of the cortical bone (Fig. 2D). However, there was no change in osteoclast surface/bone surface (Oc.S/BS) (Fig. S3A). These data indicate that S1P ablation induces osteopenia very early postnatally, as seen by the significant decrease in bone volume fraction (BV/TV) and volumetric BMD for the trabecular (Fig. S3B,C) and cortical bone (Fig. S3D-F) in P7 mice, and a drastic reduction in pMOI (a measure of resistance to torsional force), confirming the fragile nature of the bone. To analyze how these differences correlate with molecular changes in the bone, we harvested RNA from the calvaria and long bones (femur/tibia) of P10 and P21 mice, and analyzed for mature osteoblast markers by quantitative real-time polymerase chain reaction (qPCR). Both P10 (not shown) and P21 mice showed significant reductions in *type I procollagen* (*proCol1a1*), *Bglap* (*osteocalcin*) and *alkaline phosphatase* (*Alp*) expression in the calvaria (Fig. S3G) and primarily *proCol1a1* in bone (Fig. S3H). These data indicate that S1P ablation in the Osx lineage results in lower mature osteoblast numbers in the postnatal bone, and is responsible for reduced bone growth and dwarfism.

To verify that these phenotypes were caused by S1P ablation in the Osx lineage in the developing skeleton, we performed *in situ* hybridization (ISH) analysis for *Mbtps1-exon 2*, the floxed allele in S1P*^f/f^* mice. Significant S1P ablation was observed in both pre-HCs and HCs in both S1P*^+/f-Osx^* and S1P*^cko-Osx^* mice, as seen by lack of *Mbtps1-exon 2* expression (Fig. S4A-J). Notably, as Cre is expressed as a GFP-fusion protein, GFP expression is observed in pre-HCs/HCs that overlaps with zones of S1P ablation (compare Fig. S4I,J with L,M); GFP is also seen in the perichondrium (arrows, Fig. S4L,M). These data confirmed efficient S1P ablation in zones of Osx expression, and that GFP expression in our mouse model is a good surrogate for S1P ablation as reported earlier (Rodda and McMahon, 2006).

### S1P ablation in the Osx lineage delays endochondral bone development

**Fig. 3.**
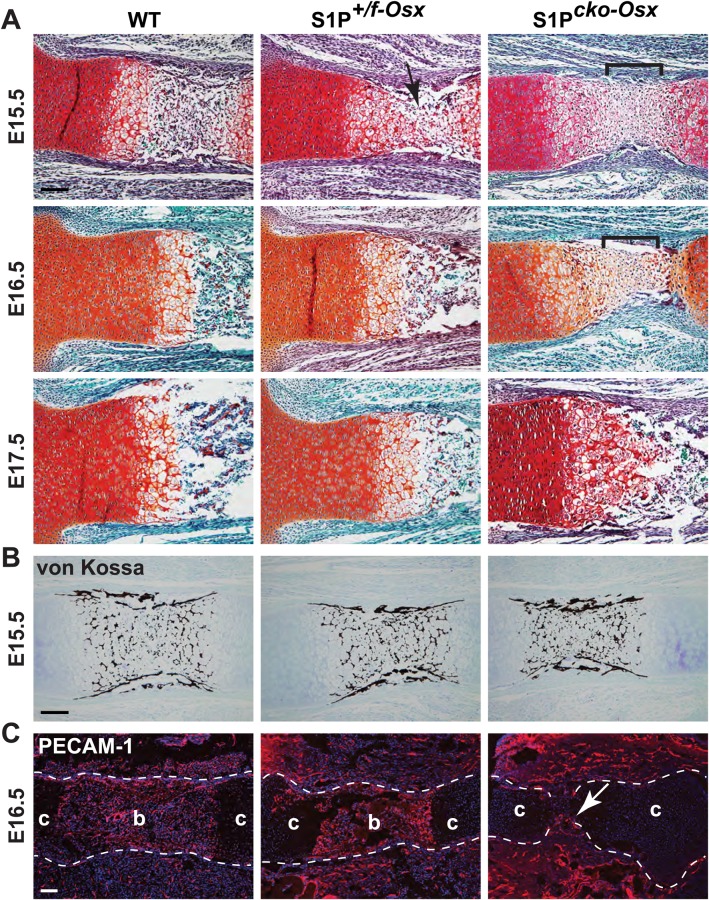
**(A) Delayed endochondral ossification in S1P*^+/f^*^-*Osx*^ and S1P*^cko-Osx^* mice.** Safranin O/Fast Green/Hematoxylin-stained femora from WT, S1P*^+/f^*^-*Osx*^ and S1P*^cko^*^-*Osx*^ at embryonic time points. Arrow points to the beginnings of vascular invasion in S1P*^+/f^*^-*Osx*^ at E15.5 but the absence of endochondral bone. Brackets denote approximate boundaries of the central expanding zone of cells in S1P*^cko^*^-*Osx*^ in the absence of endochondral bone, at E15.5 and E16.5. (B) von Kossa staining for mineralization of E15.5 femora. (C) IF for PECAM-1 at E16.5 (c, cartilage; b, bone). Arrow points to the beginning of vascular invasion in S1P*^cko^*^-*Osx*^. Scale bars: 100 μm.

To investigate why S1P ablation in the Osx lineage results in osteopenia, we first investigated embryonic bone development. At embryonic day (E) 15.5, WT mice showed characteristic development of the primary ossification center (POC) with normal endochondral bone development (Fig. 3A). In S1P*^+/f-Osx^* and S1P*^cko-Osx^* mice, however, endochondral bone development is delayed. In S1P*^+/f-Osx^* mice, only the beginning of vascular invasion is seen at E15.5 (arrow, Fig. 3A); normal endochondral bone development is observed later at E16.5. In S1P*^cko-Osx^* mice, at E15.5 and E16.5 only cartilage is seen where normally the POC would develop (brackets, Fig. 3A), with no evidence of vascular invasion; endochondral bone development is seen later at E17.5. Even though normal mineralization of the ECM is seen in all three genotypes at E15.5 (Fig. 3B), immunofluorescence (IF) analysis for the PECAM-1 antigen at E16.5 showed that vascular invasion is delayed in S1P*^cko-Osx^* (Fig. 3C).

**Fig. 4.**
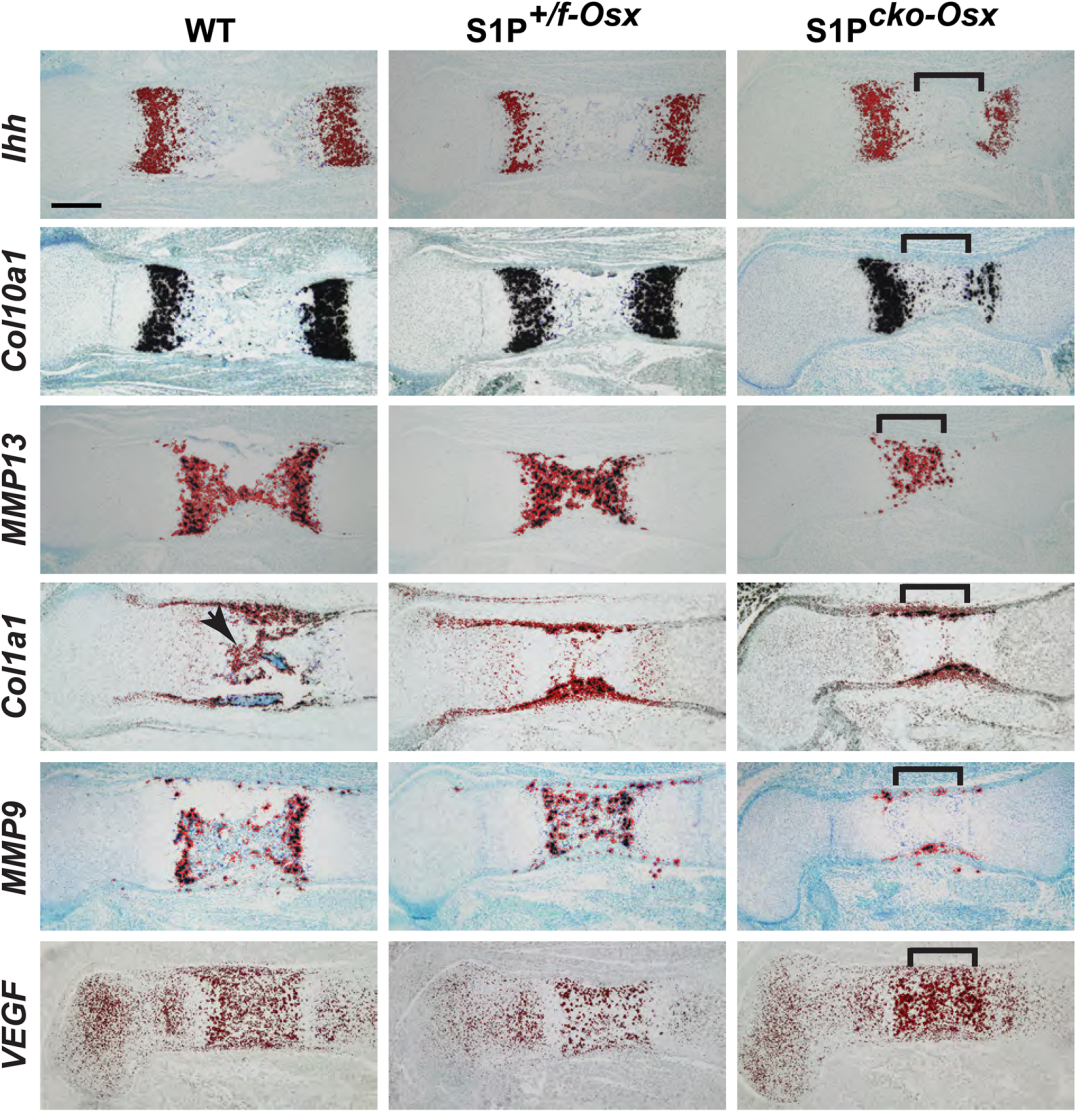
**ISH analyses of the growth plate in E16.5 femora for *Ihh*, *Col10a1*, *MMP13*, *Col1a1* and *MMP9*, and in E15.5 femora for *VEGF*.** Brackets denote approximate boundaries of the central expanding zone of cells in S1P*^cko^*^-*Osx*^ (also see Fig. 3A). Scale bars: 250 μm.

To investigate this delay, we performed ISH analysis of several molecular markers of the growth plate in E16.5 femora (Fig. 4). In S1P-ablated mice, *Ihh* (in pre-HCs) and *Col10a1* (in HCs) expression is observed in the expected zones; the thickness of the zones and expression levels of *Ihh* and *Col10a1* are similar. *Col1a1* expression is seen in the cortical bone and endochondral bone (arrow, Fig. 4) in the WT, but is primarily restricted to the cortical bones in mutant mice. In S1P*^+/f-Osx^* mice, moderate *Col1a1* expression is seen in the POC, suggesting that endochondral bone development has begun but is not as mature as in the WT. In S1P*^cko-Osx^* mice, the presumptive POC shows primarily chondrocyte-derived *MMP13* expression with faint tracings of vascular invasion by *Col1a1-*positive bone progenitors. Consequently, *MMP9* expression is seen only in the cortical bone in S1P*^cko-Osx^*, confirming its developmental lag. S1P*^cko-Osx^* mice display stronger *VEGF* expression than WT, presumably upregulated to counter the delay in vascular invasion. These data indicate that chondrocyte maturation during development is not affected, despite the absence of S1P in the Osx lineage. Morphological and ISH analysis performed in E16.5 *Osx-Cre* (*S1P^+/+^*) control mice show robust endochondral bone development similar to WT mice (Fig. S5), indicating that the defects seen in S1P*^+/f-Osx^* and S1P*^cko-Osx^* mice are due to S1P ablation.

Next, we analyzed the embryonic cartilage growth plate for Col II protein. We performed double-labeled IF for Col IIA and Col II triple helical domain (THD; the mature processed Col II in the matrix) (Fig. S6) at E16.5. Our previous studies had demonstrated that S1P ablation in the chondrocyte lineage resulted in intracellular Col II entrapment with abnormal cartilage that impeded endochondral bone development (Patra et al., 2014a, 2007). However, S1P-ablation in the Osx lineage results in cartilage matrix very similar to WT. In the resting (not shown) and proliferating zones (zones where S1P is not ablated), the cartilage matrix is identical in all three genotypes, with Col IIA (green) and Col II THD (red) distributed identically (Fig. S6A-C) with no intracellular Col II entrapment. As S1P ablation is restricted to the pre-HCs/HCs, Col II entrapment is observed in these cells but only in S1P*^cko-Osx^* (arrows, Fig. S6F). In the WT and S1P*^+/f-Osx^* mice, Col II THD (red) is seen primarily as clumps released from cells (arrows, Fig. S6D,E). In the chondrocyte-derived *MMP-13*-expressing zone present only in the S1P*^cko-Osx^* growth plate, the matrix is made primarily of Col IIA (green) with very little evidence of Col II entrapment (Fig. S6G). Double-labeled IF for pro-Col IIB and Col II THD demonstrated that the trapped collagen is pro-Col IIB, where the signals from THD (red) overlap with signals from pro-Col IIB (green) resulting in yellow colocalization signals (arrows, Fig. S6I). However, this entrapment restricted to the hypertrophic zone does not trigger apoptosis (not shown) or obliterate the growth plate. Thus, Col II entrapment is not a defining feature of this mutant phenotype, suggesting other mechanisms at play.

### S1P ablation in the Osx lineage reduces osteoblast development via a downregulation of mesenchymal progenitors

**Fig. 5.**
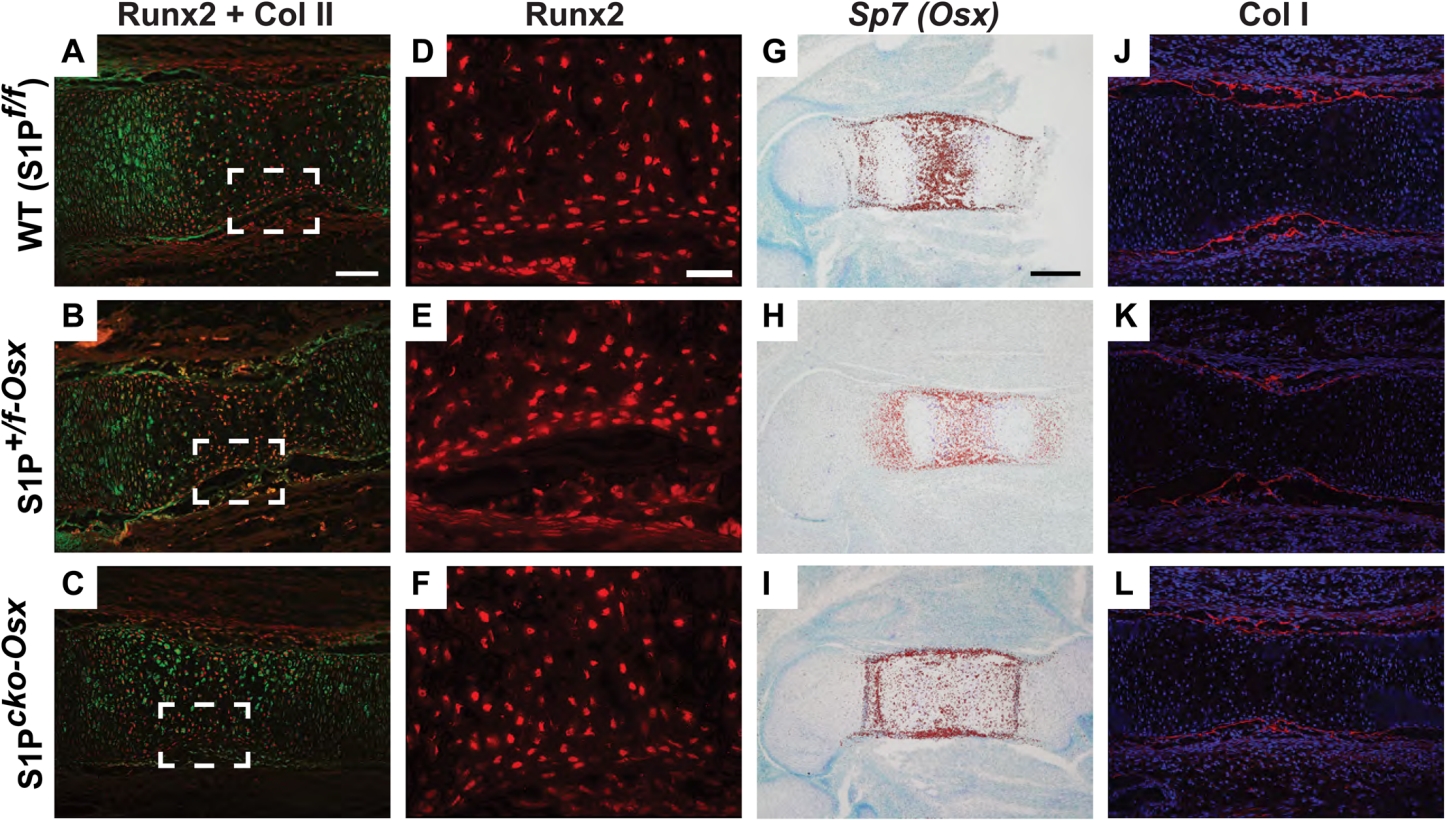
**Molecular analysis of embryonic bone development.** IF for Runx2 (red; A-F), Col II THD (green, A-C), and Col I (red, J-L) in E15.5 femora. D-F are higher magnification images of A-C (magnified region outlined in A-C; shown without signals from Col II). ISH analysis for *Sp7* (*Osx*) in E15.5 femora (G-I). Scale bars: (A-C,J-L) 100 µm; (D-F) 10 µm; (G-I) 250 µm.

Next, we studied the embryonic osteoblast lineage by analyzing for Runx2 (Ducy et al., 1997; Komori et al., 1997; Otto et al., 1997) and Osx (Nakashima et al., 2002), transcription factors that have vital roles in osteoblastogenesis. Double-labeled IF for Runx2 (red) and Col II THD (green) in E15.5 femora demonstrated that, like WT, both S1P*^+/f-Osx^* and S1P*^cko-Osx^* mice show normal Runx2 expression in pre-HCs/HCs of the growth plate (Fig. 5A-C) and in the perichondrium (Fig. 5D-F). Likewise, ISH analysis for *Sp7* (*Osx)* in E15.5 femora demonstrated normal *Sp7* expression in all three genotypes (Fig. 5G-I), though the expression pattern is different, reflecting their developmental lag (Fig. 3A). Consequently, normal type I collagen protein (Col I) deposition is seen in the bone collar of S1P*^+/f-Osx^* and S1P*^cko-Osx^* mice as in the WT (Fig. 5J-L). We also investigated components of the Wnt/β-catenin signaling pathway that have important roles in osteoblast differentiation (Hill et al., 2005; Hu et al., 2005; Rodda and McMahon, 2006). Using protein lysates made from the hind limbs of E14.5 mice, we investigated phosphorylation levels of Akt and GSK-3β and total β-catenin levels in immunoblots. Identical levels of Akt and GSK-3β phosphorylation and β-catenin levels are observed in protein lysates from all three genotypes (Fig. S7) indicating normal Wnt/β-catenin signaling in mutant mice.

**Fig. 6.**
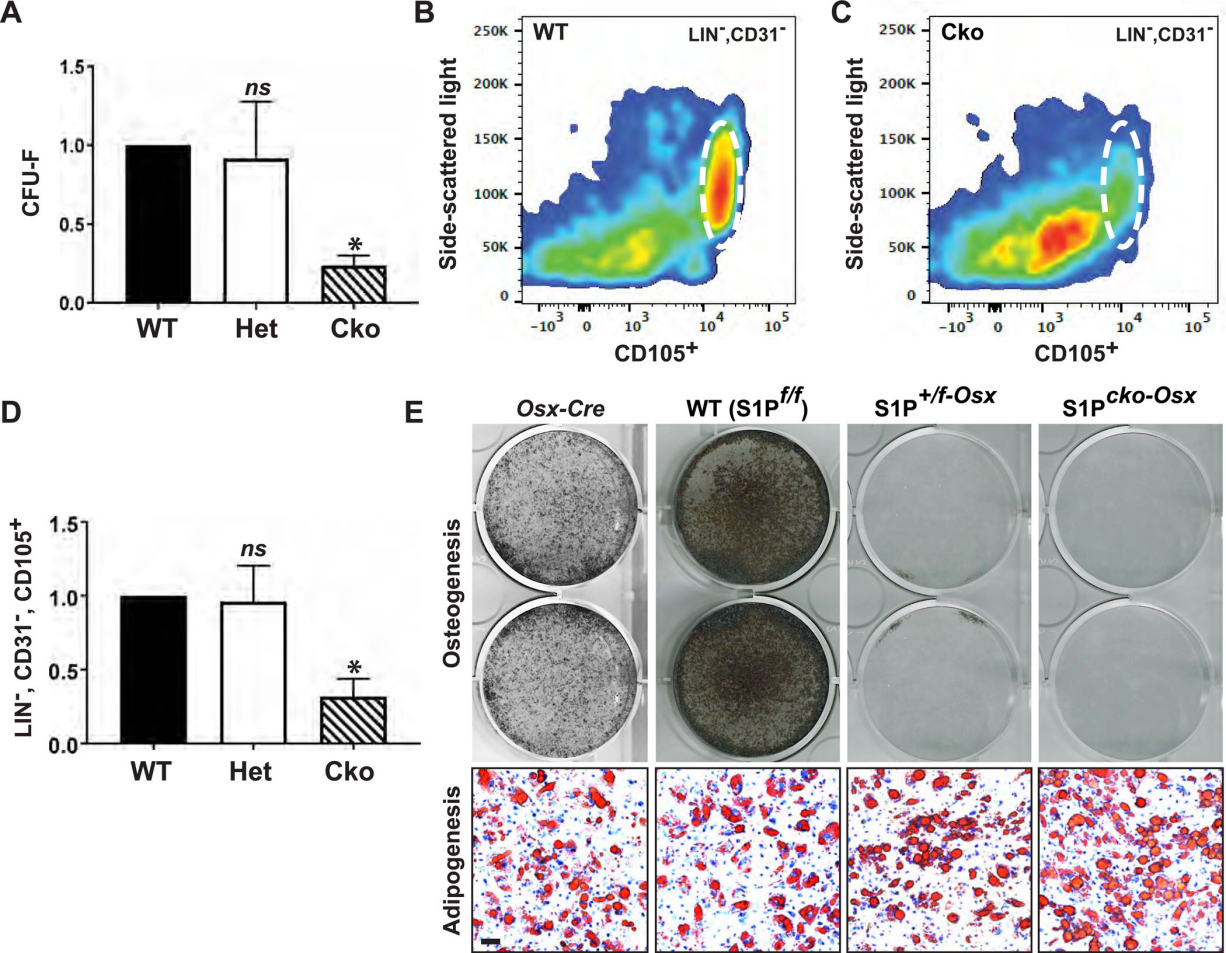
**Downregulation of SSCs in S1P*^cko-Osx^*.** (A) Enumeration of CFU-F in P21 WT, S1P*^+/f-Osx^* (Het) and S1P*^cko-Osx^* (Cko) mice. Data shown as percent of WT to normalize between biological repeats (mean±s.d.; *P<0.005 or not significant (*ns*) when compared to WT; *N*=3). (B,C) Scatter plot of FACS analysis for SSCs from P21 WT (B) and S1P*^cko-Osx^* (C). LIN^−^, CD31^−^, CD105^+^ cells are demarcated by an ellipse. A typical result is shown. (D) Enumeration of LIN^−^, CD31^−^, CD105^+^ SSCs. Data shown as percent of WT (mean±s.d.; *P<0.001 or *ns* when compared to WT; *N*=4). (E) *In vitro* osteogenic and adipogenic differentiation assays with BMSCs from P21 mice. Typical results are shown from *N*=3.

Next, we investigated if skeletal progenitors were affected. The bone marrow is a niche for mesenchymal-derived skeletal stem cells (SSCs) and recent studies have identified specific SSCs with temporal and lineage-specific contributions to skeletal development (Chan et al., 2015; Worthley et al., 2015). Besides, Osx is active in bone marrow stromal cells (BMSCs) (Chen et al., 2014; Mizoguchi et al., 2014), suggesting that S1P ablation in these cells could affect the postnatal bone marrow compartment and, consequently, skeletal development. Therefore, we first analyzed the ability of BMSCs from P21 WT, S1P*^+/f-Osx^* (Het) and S1P*^cko-Osx^* (Cko) mice to form colony-forming unit-fibroblasts (CFU-F), a measure of mesenchymal progenitors in the bone marrow. While the WT and S1P*^+/f-Osx^* mice showed similar CFU-F capabilities, this capability was significantly reduced in S1P*^cko-Osx^* mice (Fig. 6A). Next, we analyzed if the downregulation in CFU-F correlates with a downregulation of SSCs. SSCs have been defined as CD105-expressing (CD105^+^) cells that are triple negative for CD45, Ter-119 and CD31 (Chan et al., 2015; Worthley et al., 2015). CD45 is expressed on most hematopoietic cells, except maturing erythroid cells that express Ter-119; CD31 is expressed on endothelial cells. Using fluorescent-conjugated antibodies for CD45, Ter-119, CD31 and CD105, we analyzed LIN^−^ (CD45^−^, Ter-119^−^), CD31^−^, CD105^+^ cells in the bone marrow/collagenase-digested bones from P21 mice by fluorescence-activated cell sorting (FACS) analysis. In parallel to that seen for CFU-F, WT and S1P*^+/f-Osx^* mice showed similar levels of LIN^−^, CD31^−^, CD105^+^ SSCs, while S1P*^cko-Osx^* mice demonstrated a drastic reduction in these SSCs (Fig. 6B-D). Given the similar numbers of SSCs in WT and S1P*^+/f-Osx^* mice, it was puzzling that S1P*^+/f-Osx^* mice are smaller than WT or *Osx-Cre* mice. Therefore we analyzed the capacity of BMSCs for osteogenic differentiation *in vitro* (Fig. 6E). WT mice showed strong osteogenic differentiation *in vitro* as seen by the presence of mineralized bone nodules. Control *Osx-Cre* (*S1P^+/+^*) mice also demonstrated osteogenic differentiation (though this was reduced when compared to WT). In contrast, neither S1P*^+/f-Osx^* or S1P*^cko-Osx^* mice were able to demonstrate osteogenic differentiation. To analyze if this was a generic progenitor issue, we analyzed if BMSCs were capable of undergoing adipogenesis *in vitro* (Fig. 6E). Interestingly, both S1P*^+/f-Osx^* and S1P*^cko-Osx^* mice demonstrated adipogenic differentiation similar to *Osx-Cre* and WT mice. These data indicate a specific requirement for S1P not only to maintain the SSC population but also for their osteogenic differentiation.

**Fig. 7.**
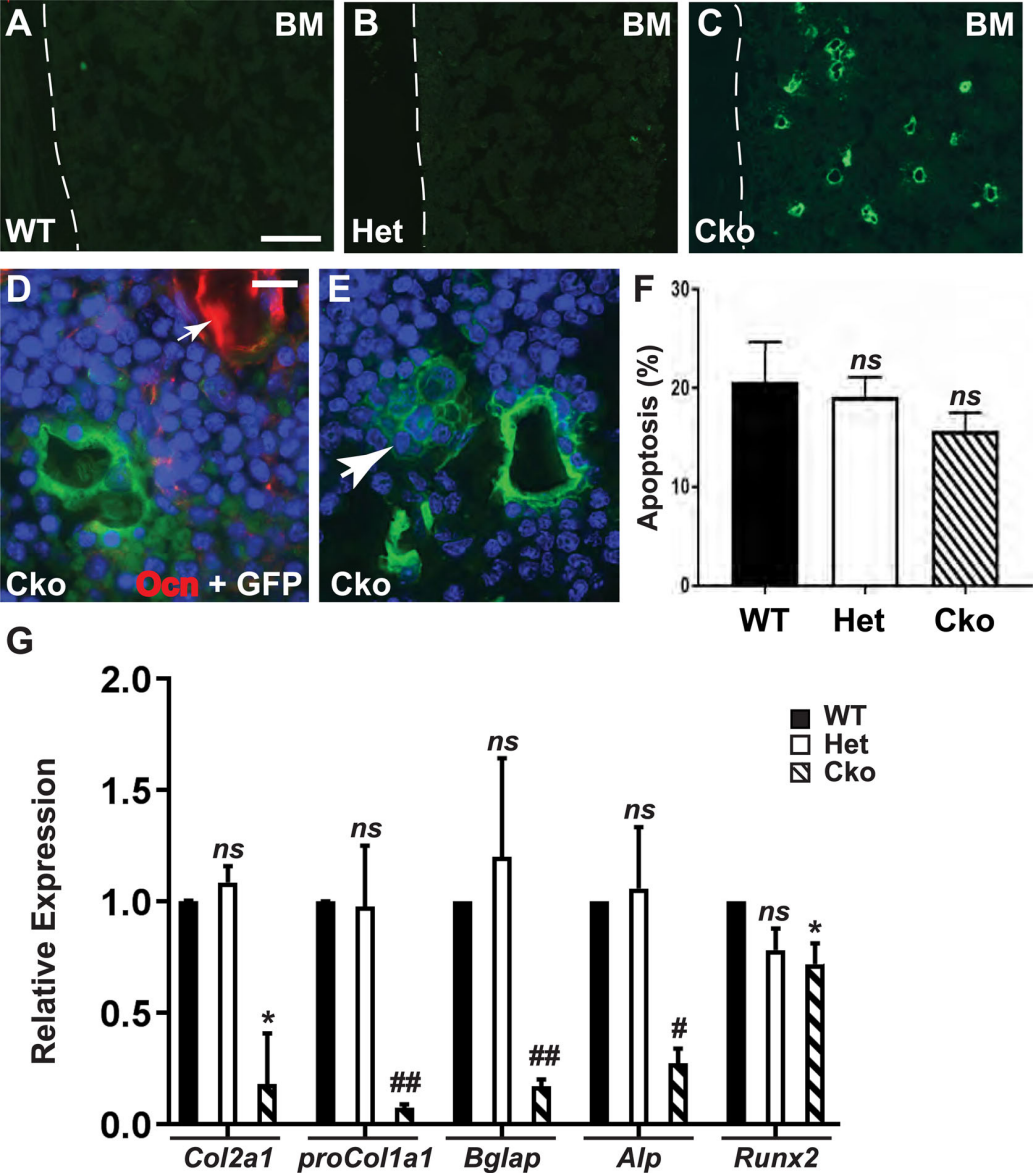
**Analysis of the bone marrow compartment.** (A-C) GFP^+^ cells in the bone marrow of P21 S1P*^cko-Osx^* (Cko) femur, absent in WT or Het (also see Fig. S8). Dashed lines show junction of bone marrow with cortical bone. (D) IF for osteocalcin (Ocn, red) in P21 Cko. (E) GFP^+^ rosettes (arrow) in the Cko bone marrow (blue, DAPI-stained nuclei). (F) FACS analysis for apoptosis (% cells labeled with Annexin V conjugate) in cells from P21 bone marrow (mean±s.d.; *N*=3). (G) qPCR analysis for *Col2a1*, *proCol1a1*, *Bglap*, *Alp* and *Runx2* with RNA harvested from P21 bone marrow (mean±s.d.; *N*=3; *P<0.05, ^#^*P*<0.005, ^##^*P*<0.001, or *ns* when compared to WT). Scale bars: (A-C) 100 µm; (D,E) 10 µm.

Interestingly, we found the presence of many GFP-expressing (GFP^+^) cells in the bone marrow of S1P*^cko-Osx^* (Cko) mice, which are absent in WT or Het mice (Fig. 7), or age-matched *Osx-Cre* mice (Fig. S8). Absence of GFP^+^ cells in the WT (which do not carry the *Osx-Cre* transgene) is expected. But GFP^+^ cells are missing in the bone marrow of S1P*^+/f-Osx^* (Het) (Fig. 7) or *Osx-Cre* mice (Fig. S8) that carry the transgene, even though these mice show GFP^+^ chondrocytes in the hypertrophic zone of the growth plate (arrows, Fig. S8). These GFP^+^ cells do not express osteocalcin (Ocn, a marker of mature osteoblast), though Ocn was detected on bone surfaces (arrow, Fig. 7D), and often appeared arranged in a rosette-like structure in the bone marrow (arrow, Fig. 7E). To study if the absence of S1P induces apoptosis in bone marrow constituents, we analyzed apoptosis by FACS using a fluorescent-labeled Annexin V conjugate, which detects apoptosis-induced externalization of phosphatidylserine on the cell surface. However, no more apoptosis was detected in the Cko bone marrow than that seen in WT or Het (Fig. 7F). These GFP^+^ cells in the Cko bone marrow indicate that these cells are in the Osx lineage and that their detection is possible due to an arrest in differentiation, presumably to the osteoblast lineage. The absence of these cells in the WT, Het and *Osx-Cre* mice indicates conditions favorable for SSC maintenance and maturation in these mice. qPCR analysis of RNA harvested from the bone marrow showed significant decrease in the expression of *proCol1a1*, *Bglap*, *Alp*, *Runx2* and also *Col2a1* (a marker for osteochondroprogenitors) in S1P*^cko-Osx^* when compared to WT and S1P*^+/f-Osx^* mice (Fig. 7G), an observation that parallels CFU-F and SSC levels seen in S1P*^cko-Osx^* mice. These data collectively indicate that S1P ablation in the Osx lineage adversely affects bone development by downregulating osteoprogenitors and their differentiation.

### S1P ablation in the Osx lineage reduces the postnatal growth plate

**Fig. 8.**
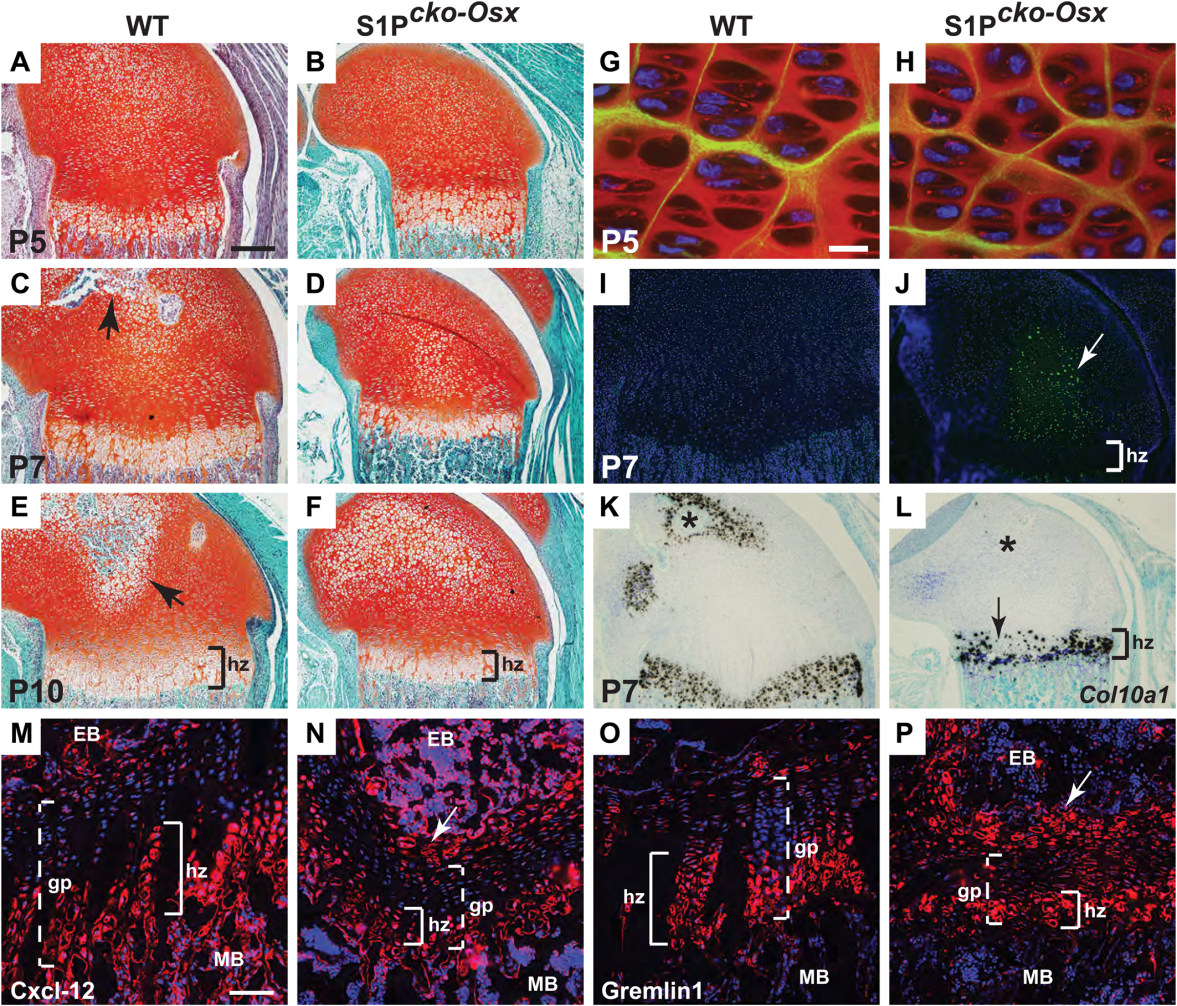
**Downregulation of the chondrocyte lineage in S1P*^cko-Osx^* mice.** (A-F) Safranin O-stained sections of the growth plate and epiphyseal cartilage in P5 (A,B), P7 (C,D) and P10 (E,F) femora (distal end). Arrows in C and E point to the developing SOC. (G,H) Double-labeled IF for Col II THD (red) and Col IIA (green) in P5 femora of WT (G) and S1P*^cko-Osx^* (H). (I,J) TUNEL assay in P7 WT and S1P*^cko^*^-*Osx*^ distal femora. Apoptosis (arrow) is seen in green against DAPI-stained blue nuclei. (K,L) ISH analyses for *Col10a1* in P7 femoral growth plate in WT (K) and S1P*^cko^*^-*Osx*^ (L). Asterisks mark the presumptive SOC. Arrow points to regions of missing *Col10a1* expression in S1P*^cko-Osx^* hypertrophic zone. (M-P) IF for Cxcl-12 (red) (M,N) and Gremlin1 (red) (O,P) in P21 mice. Arrow points to incomplete removal of cartilage from the epiphysis. (gp, growth plate; hz, hypertrophic zone; EB, epiphyseal bone; MB, metaphyseal bone). Scale bars: (A-F,I-L) 250 μm; (G, H) 10 μm; (M-P) 100 μm.

SSCs that contribute to the osteoblast lineage, downregulated in S1P*^cko-Osx^* mice, also contribute to the chondrocyte lineage (Chan et al., 2015; Worthley et al., 2015). Therefore we investigated if S1P ablation affects the postnatal cartilage and growth plate (Fig. 8). The epiphyseal cartilage and growth plate in WT, S1P*^+/f-Osx^* and S1P*^cko-Osx^* mice look identical to each other up to 5 days postnatally. The WT and S1P*^+/f-Osx^* mice showed identical phenotypes in this analysis and therefore only WT and S1P*^cko-Osx^* are shown. At P5, with the exception of the bone width that is smaller in S1P*^cko-Osx^* mice, the growth plate and epiphyseal cartilage are indistinguishable (Fig. 8A,B) (P5 S1P*^cko-Osx^* mice are however smaller than WT, Fig. S2B). The WT shows incipient secondary ossification center (SOC) at P7 that develops further at P10 (arrows, Fig. 8C,E). However, the SOC is completely absent even at P10 in S1P*^cko-Osx^* mice; it develops very slowly and even at P21 the epiphyseal cartilage in the SOC is not completely replaced by trabecular bone (Fig. S8C). The hypertrophic zone is smaller in S1P*^cko-Osx^* (bracket, Fig. 8F) when compared to WT (Fig. 8E).

To investigate if this was induced by abnormal Col II deposition, we analyzed the epiphyseal cartilage by double-labeled IF for Col IIA and Col II THD as above. No marked differences between WT and S1P*^cko-Osx^* mice is seen at any stage in Col II deposition (P5 shown as an example, Fig. 8G,H). However, S1P*^cko-Osx^* showed extensive chondrocyte apoptosis in the epiphyseal cartilage at P7 (arrow, Fig. 8J), just above the hypertrophic zone of the growth plate. In previous studies, apoptosis was effected by abnormal pro-Col IIB entrapment that induced UPR and delayed SOC formation (Patra et al., 2011). However, no abnormal Col II entrapment was observed at P5 (Fig. 8G,H) (or other stages, not shown). Chondrocyte apoptosis overlapped with columnar cells of the growth plate (which normally mature to HCs). ISH analysis showed that while *Col10a1* expression is observed in the presumptive SOC (asterisk) and is uniform in the WT growth plate (Fig. 8K), it is completely missing in the presumptive SOC (asterisk) in S1P*^cko-Osx^* and is patchy in the hypertrophic zone with large areas missing *Col10a1* expression (arrow, Fig. 8L). Apoptotic death of chondrocytes would preclude their maturation to HCs that would keep this zone small in S1P*^cko-Osx^*. Recent studies demonstrated that Gremlin1^+^ cells (Worthley et al., 2015) and CAR (Cxcl12-abundant reticular) cells are a source of chondrocyte progenitors (Omatsu et al., 2010). To investigate growth plate chondrocytes further in light of the decline of the hypertrophic zone and non-uniform expression of *Col10a1*, we analyzed Cxcl-12 and Gremlin1 expression in the P21 growth plate. Both WT and S1P*^cko-Osx^* show expression for Cxcl-12 and Gremlin1 in HCs, characteristically missing in younger, proliferative chondrocytes (Fig. 8M-P). Similar to *Col10a1* expression, the WT has a bigger zone of Cxcl-12- (Fig. 8M) and Gremlin1-expressing cells (Fig. 8O) than S1P*^cko-Osx^*. Like the hypertrophic zone (hz) in P7 mice (Fig. 8F,L), Cxcl-12- (Fig. 8N) and Gremlin1-expressing (Fig. 8P) zones are greatly reduced in S1P*^cko-Osx^*. These observations confirm the significant loss of postnatal growth plate chondrocytes and of the hypertrophic zone in these mice. S1P*^cko-Osx^* show a larger number of Cxcl-12- and Gremlin1-expressing chondrocytes at the junction of the epiphyseal bone with the growth plate (arrow, Fig. 8N,P) than the WT at this junction. This expression is in chondrocytes left over from the incompletely replaced epiphyseal cartilage in S1P*^cko-Osx^* that is otherwise efficiently replaced by bone in the WT (Fig. S8C).

## DISCUSSION

S1P has emerged as a critical regulator of mammalian skeleton development. It is a fundamental component of the regulated intramembrane proteolysis system where it plays an active role along with S2P (*Mbtps2*) protease in processing precursor proteins to their active form (Brown and Goldstein, 1999; Eberlé et al., 2004). Even though S1P mutations in humans are yet to be reported, mutations in SREBP-2 (a major S1P/S2P substrate) are linked to osteoarthritis pathogenesis in humans (Kostopoulou et al., 2012), and mutations in S2P are linked to osteogenesis imperfecta (Lindert et al., 2016). S1P therefore participates in a major homeostatic pathway needed for skeletal development and maintenance. In this study we demonstrated that S1P has a direct role in bone development. Our studies on S1P in the Osx lineage have identified a cellular mechanism where S1P is required to maintain LIN^−^, CD31^−^, CD105^+^ SSCs of the skeletal mesenchyme, which are precursors for chondrocyte and osteoblast lineages and for osteoblast differentiation.

S1P ablation in the Osx lineage results in short-statured mice, for which the extensiveness of their short stature correlates with the degree of ablation, with the heterozygous S1P*^+/f-Osx^* showing an intermediate size between the WT/*Osx-Cre* and S1P*^cko-Osx^*. S1P*^cko-Osx^* mice suffer from osteochondrodysplasia as both chondrocyte and osteoblast lineages are affected, contributing to declining bone growth. Part of the reason could be due to a lack of OASIS activity. OASIS, an established substrate for S1P, is a transcription factor for *Col1a1* and *Col1a2* genes (Murakami et al., 2009). However, while *OASIS*^−/−^ mice are also smaller than WT, this reduction in size is not as severe as seen in S1P-ablated mice. Furthermore, qPCR analysis of RNA isolated from the calvaria of *OASIS*^−/−^ mice demonstrated a reduction only in *Col1a1* and *Col1a2* expression, while *Bglap* and *Alp* were upregulated. However, S1P-ablated mice show a drastic decrease in size that can be discerned as early as P5 and a reduction in the expression of *proCol1a1*, *Bglap* and *Alp*. Thus, unlike *OASIS*^−/−^ mice, S1P ablation in the Osx lineage mediates a global downregulation of the osteoblast lineage, consistent with a role in maintaining SSC population, and not via OASIS functions.

During embryonic bone development, at E13.5, before the formation of bone marrow cavity, Osx^+^ progenitors from the perichondrium migrate into the POC, along with blood vessels (Maes et al., 2010; Mizoguchi et al., 2014). Because of the direct association between vascular invasion and invading osteoprogenitors that initiates POC development, a defect in either of these two components could induce a delay in endochondral bone development. A decrease or a defect in perichondrial osteoprogenitors is plausible due to S1P ablation in these cells (due to *Osx-Cre* activity in perichondrium deduced from the presence of GFP^+^ cells). A decrease/defect in perichondrial osteoprogenitors could induce a delay, owing to lack of adequate numbers of functional progenitors required to set up the POC, or to crosstalk with the vascular invasion mechanism. A delay in vascular invasion could be induced by the entrapped pro-Col IIB in the hypertrophic zone in S1P*^cko-Osx^* where vascular invasion takes place. S1P*^cko^* mice (*Col2-Cre* driven) suffered from a complete lack of vascular invasion where pro-Col IIB entrapment was absolute (Patra et al., 2014a, 2007). This suggests that clearing of the entrapped pro-Col IIB in this zone by UPR may be necessary before vascular invasion can begin. In contrast to S1P*^cko^* mice, the limited pro-Coll B entrapment in S1P*^cko-Osx^* allows the removal of the trapped pro-Col llB without inducing apoptosis. However, a delay in vascular invasion is also seen in the heterozygous S1P*^+/f-Osx^* mice, which do not exhibit any pro-Col IIB entrapment. Thus, S1P*^+/f-Osx^* mice may suffer from only a lack of adequate Osx^+^ perichondrial progenitor functions, while a defect in Osx^+^ perichondrial progenitors coupled to the entrapped pro-Col IIB may induce a more pronounced delay in vascular invasion seen in S1P*^cko-Osx^* mice.

Lineage tracing experiments have demonstrated the presence of temporally distinct fetal, perinatal and adult Osx^+^ bone progenitors (Maes et al., 2010; Mizoguchi et al., 2014). Fetal perichondrial Osx^+^ progenitors contribute significantly to perinatal (∼8 day old mice) bone and BMSCs, but not to adult bone or bone marrow. However, perinatal Osx^+^ progenitors contribute to perinatal bone development and to long-lived stromal cells that contribute to osteolineages in growing and adult mice. This is in good standing with our observations that the biggest differences in bone development on S1P ablation in the Osx lineage is seen very early perinatally, a consequence of S1P ablation in fetal Osx^+^ perichondrial progenitors. While the mice are similar in size when born, P5 S1P*^cko-Osx^* are easily detected due to their stunted growth, which becomes more pronounced at P7. Postnatally, the presence of GFP^+^ cells in the bone marrow of S1P*^cko-Osx^* mice (missing in *Osx-Cre*/WT/S1P*^+/f-Osx^*) and its size difference (much larger) from other cells in the bone marrow indicate that they are not of hematopoietic lineage, but a subpopulation of BMSCs. The expression of GFP indicates that these are Osx^+^ osteoprogenitors arrested at a specific stage of osteoblastogenesis that requires S1P. This rationale is further supported by the inability of BMSCs to differentiate into osteoblasts *in vitro*, coupled with an inability to detect osteoblasts/bone lining cells *in vivo*. The similarity in the expression profile for *Col2a1*, *proCol1a1*, *Bglap* and *Alp* between the WT and S1P*^+/f-Osx^*, along with similar values for CFU-F and SSCs and the absence of GFP^+^ cells indicate that their osteoblast differentiation program is normal, which is in contrast to the homozygous knockout S1P*^cko-Osx^* mice. However, *in vitro*, S1P*^+/f-Osx^* differs from WT in its inability to exhibit osteogenic differentiation, an aspect that presumably contributes to its smaller skeletal size than the WT. S1P*^cko-Osx^* mice suffer from the double disadvantage that both bone (reduced CFU-F and SSCs and defective osteogenic differentiation) and chondrocyte (reduced growth plate thickness and hypertrophic zone) lineages are downregulated. As HCs can transdifferentiate into osteoblasts, the reduction of the hypertrophic zone in S1P*^cko-Osx^* growth plate would suggest a reduction in the number of HCs transdifferentiating to osteoblasts and therefore a reduction of chondrocyte contribution to osteoblast development. Thus, while there are different origins for postnatal bone, the bone formed in S1P*^cko-Osx^* mice has drastically reduced contribution of Osx^+^ osteoprogenitors and, conceivably, HCs-derived osteoblasts.

S1P*^cko^* mice are unable to make any endochondral bone (Patra et al., 2007). Though it did not prevent endochondral bone formation, S1P ablation in the Osx lineage decreased overall bone development drastically by downregulating SSC population and preventing their differentiation. Thus, in S1P*^cko^*, it is possible that besides adversity from the abnormal cartilage, a defect in osteoprogenitors also contributed to the lack of endochondral bone formation. As most osteoblasts, CAR and CFU-F cells are descendants of *Col2-Cre^+^* cells (Ono et al., 2014); S1P ablation by *Col2-Cre* may induce a stronger mutant phenotype as it targets a more diverse population of osteoprogenitors than *Osx-Cre*. As progenitors are derived from stem cells and *Col2a1* is expressed in bone marrow osteoprogenitors and not strictly in chondrocytes (Szabova et al., 2009; Wang et al., 2011), the reduction in *Col2a1* expression observed in the S1P*^cko-Osx^* bone marrow presumably reflects the observed reductions in CFU-F and SSCs in these mice. These observations lend credence to the requirement of S1P in maintaining the SSC population in the murine bone marrow. S1P ablation in the Osx lineage, however, did not affect adipogenesis, indicating that this ablation did not interfere with other progenitor functions in the bone marrow. As S1P is not a secreted protein (Pullikotil et al., 2007), this observation may suggest that S1P ablation in the Osx lineage in the bone marrow does not cause non-cell autonomous mutational effects, in agreement with mosaic analysis in zebrafish (Schlombs et al., 2003). How S1P maintains the SSC population is not known as we have not yet identified a molecular target, but our studies clearly indicate a requirement for S1P in the Osx lineage for normal bone development.

The development of scoliosis in S1P*^cko-Osx^* mice very early postnatally is reminiscent of adolescent idiopathic scoliosis (AIS) in humans (Lonstein, 1994). The etiology of scoliosis remains unknown although genetic predisposing factors likely contribute to this multifactorial disease. Scoliosis may arise in S1P*^cko-Osx^* due to the reduction in BMD and consequent weak bones that cannot resist spine bending and/or twisting. Poor bone quality as an etiological factor in scoliosis remains controversial, but low BMD is a generalized phenomenon and a systematic disorder in AIS. The prevalence of AIS with osteoporosis is approximately 20-38% (Li et al., 2008). The associated osteochondrodysplasia and low BMD seen in S1P*^cko-Osx^* mice suggests that it mimics the chondrodysplasia-related scoliosis seen in humans (Mason et al., 2002). Studies show that scoliosis can manifest due to gene deletions in osteochondroprogenitors; for example, the *Col2Cre*-directed deletion of *Neurofibromatosis type I* gene (Wang et al., 2011) or *Src homology-2* gene (Kim et al., 2013). Thus, S1P-ablated mouse models are uniquely placed to investigate the molecular mechanisms for skeletal dysplasias and associated spine abnormalities and provide a genetic starting point to study and identify the molecular framework for investigating SSC differentiation to osteoblasts.

## MATERIALS AND METHODS

### Ethics statement

All mouse procedures were performed in accordance with the National Institutes of Health (NIH) Guide for the Care and Use of Laboratory Animals, using vertebrate animals/ethics protocols reviewed and approved by the Animal Studies Committee at Washington University School of Medicine.

### S1P ablation in the osterix lineage in mice

For mice with S1P ablation in the osterix (Osx) lineage, S1P*^f/f^* mice (mice homozygous for the floxed *exon 2* of *Mbtps1*; in C57BL/6) (Yang et al., 2001) were bred with *Osx1-GFP::Cre* [Cre recombinase expressed as a fusion protein with GFP from the *Sp7 *(*Osx*)** promoter] (Rodda and McMahon, 2006) transgenic mice in the C57BL/6J strain to produce S1P*^+/f^*;*Osx-Cre* mice (mice heterozygous for S1P*^flox^* allele with *Osx-Cre* transgene). The heterozygous S1P*^+/f^*;*Osx-Cre* (S1P*^+/f-Osx^* or Het) mice were bred with S1P*^f/f^* mice to generate mice with homozygous deletion of S1P (S1P*^f/f^*;*Osx-Cre* or S1P*^cko-Osx^* or Cko) in the Osx lineage. The *Osx1-GFP::Cre* (henceforth referred to as *Osx-Cre*) mice can be regulated by the tetracycline transactivator (tTA) that renders the *Osx* promoter inactive in the presence of doxycycline. In our studies, the mice were never fed doxycycline and therefore the *Osx-Cre* transgene was never temporally regulated by doxycycline. Genotypes were verified by PCR analysis of tail-derived DNA. As *Osx-Cre* mice have skeletal defects (Huang and Olsen, 2015), they were used as controls initially to confirm that the mutant phenotypes were caused by S1P ablation; otherwise, the heterozygote mice positive for *Osx-Cre* served as appropriate controls. Males and females showed identical phenotypes. However S1P*^cko-Osx^* mice have difficulty surviving beyond weaning and require special and accessible food supplies to prolong their survival further for a short time (typically 7-8 days) post-weaning.

### μCT

For μCT analyses, mice were skinned, eviscerated, and fixed in 10% neutral buffered formalin for 24-48 h, washed and stored in 70% ethanol. Skeletal elements were scanned in a VivaCT 40 scanner (Scanco Medical AG, Bruttisellen, Switzerland) at medium to high resolution and tube settings of 55 kV peak of energy, 145 μA of current with an integration time of 300 ms. Segmentation was performed to distinguish high density (bone) from low density (soft tissue/cartilage/growth plate) areas. Images from individual scan slices or reconstructed bones were captured as TIFF images. Morphometric measurements were calculated using 35-50 scan slices and the manufacturer's 3D analysis tools, and is based on the direct method of calculation (Hildebrand et al., 1999). Bone mineral density (BMD) heat maps were generated using OsiriX (Pixmeo SARL, Geneva, Switzerland) software and Jet color scheme, where yellowish-orange represents highest BMD and blue the lowest.

### Dynamic histomorphometry

P21 mice were injected intraperitoneally with the fluorochrome calcein green (Sigma-Aldrich; 10 mg/kg) followed by alizarin complexone (Sigma-Aldrich; 30 mg/kg) 5 days later. Following alizarin administration, the hind limbs were harvested 2 days later and embedded in methyl methacrylate (Baron et al., 1983). Sections (10 μm thick) were visualized by fluorescent microscopy for calcein/alizarin incorporation. In these young mice, calcein-alizarin double labeling was observed primarily in the cortical bones. Dynamic histomorphometric measures such as mineral apposition rate (MAR), bone formation rate/bone surface (BFR/BS) and osteoclast surface/bone surface (Oc.S/BS) for the endosteal surface of the cortical bone was analyzed using Osteo II (BIOQUANT, Nashville, TN, USA).

### qPCR analysis

For qPCR analysis, femur and tibia from hind limbs or calvariae were harvested, muscle and other tissues removed, and the bone marrow flushed out and analyzed separately. Bones and calvariae were pulverized in a Mikro-Dismembrator U (B. Braun Biotech International Melsungen, Germany) and RNA harvested using Trizol according to the manufacturer's recommended protocol and column purified by RNeasy mini kit (Qiagen). RNA (1 μg) was reverse transcribed into cDNA using a RT^2^ First Strand kit (Qiagen) and the cDNA used for qPCR analysis using SYBR Green primer sets, 2X SYBR Green mix (Life Technologies/Applied Biosystems) using standard protocols and the relative amount of mRNA calculated using the comparative C_T_ method. As recommended by the MIQE standards for qPCR, normalization was performed using both murine *Gapdh* and *18S* genes. Both control genes yielded identical results. SYBR Green primer sets for the murine *18S* (Yoda et al., 2010), *Gapdh*, *proCol1a1* (*type I procollagen, alpha1*), *Bglap* (*osteocalcin*) and *alkaline phosphatase *(*Alp*)** are as previously reported (Zhang et al., 2011).

### CFU-F assay

For CFU-F assays, the bone marrow was harvested from both hind limbs of P21 mice and red blood cells (RBCs) lysed using RBC lysis buffer (Sigma-Aldrich). Nucleated cells (1×10^6^) were then plated in T75 cm^2^ flasks and cultured for 2 days in α-MEM with 20% fetal bovine serum (FBS) and 2% penicillin/streptomycin. After 2 days, the medium was removed completely to remove dead cells, fresh medium added and cultured for a further 8 days, at which point colonies were stained with Methylene Blue. Only colonies that had at least 50 cells were counted as a viable colony.

### FACS analysis for CD105^+^ SSCs

Analysis of LIN-negative (CD45^−^, Ter-119^−^), endothelial-negative (CD31^−^), CD105^+^ SSCs were done as described (Worthley et al., 2015). Briefly, long bones (striped of skin and muscle tissue) from the hind limbs of P21 mice were harvested, crushed in a mortar and pestle with PBS and filtered using a 50 μm filter. The sediment in the filter was digested with collagenase type IV (1.7 mg/ml) (Thermo Fisher Scientific) for 10 min at 37°C and mixed with filtrate from above. After several washings in PBS (with 0.1% bovine serum albumin), the cells were incubated with fluorescent-conjugated antibodies to CD45 (PE-Cy7) (552848, BD Pharmingen, San Jose, CA, USA), Ter-119 (PE-Cy7) (25-5921, eBioscience, San Diego, CA, USA), CD31 (BV421) (BD Pharmingen, 563356), and CD105 (Alexa Fluor 647) (562761, BD Pharmingen) and FACS analysis performed on BD FACSAria following standard protocols. In gating analysis (performed by FlowJo_V10; www.flowjo.com), single cells were gated negatively for PE-Cy7 signals (CD45- and Ter-119-expressing) to remove hematopoietic and erythrocyte cells, followed by negative gating for BV421 signals (CD31-expressing) to remove endothelial cells from the population of CD105^+^ cells (Alexa Fluor 647 signals).

### *In vitro* osteogenic and adipogenic differentiation assays

Bone marrow was harvested from the hind limbs of P21 mice and the resulting cell population obtained after lysis of RBCs were cultured in a single well of a six-well plate in α-MEM with 20% FBS and 2% penicillin/streptomycin until confluent. Once confluent, cells were passaged into a 6 cm dish and cultured until confluent. The cells were then cultured in two wells of a six-well plate, grown until confluent at which point osteogenic differentiation media (α-MEM with 10% FBS, 2% penicillin/streptomycin,10 mM β-glycerophosphate, 50 μg/ml of ascorbic acid) was added and cultured for 14 days. Osteogenic differentiation was assessed by silver staining of the mineralized matrix by the von Kossa method. For adipogenic differentiation, cells obtained after lysis of RBCs were cultured in a single well of a six-well plate in complete MesenCult™ Expansion medium (STEMCELL Technologies, Vancouver, Canada) at 37°C under hypoxic conditions until 80-90% confluent. The medium was then replaced with complete MesenCult™ Adipogenic Medium (STEMCELL Technologies), and the cells were incubated further at 37°C in hypoxic conditions with a change in medium every 3 days for a total of 14 days. Adipogenic differentiation was analyzed by staining for lipid droplets by Oil Red O.

### ISH analysis

ISH analyses were performed on 5-µm paraffin-embedded sections as described previously using ^35^S-labeled riboprobes (Long et al., 2001; Patra et al., 2007). ISH images were viewed with a BX51 microscope (Olympus, Waltham, MA, USA) and images captured with a digital camera (DP70; Olympus) using DP controller software (Olympus). Images of hybridization signals were pseudo-colored red and superimposed on Toluidine Blue-counterstained images using Photoshop (Adobe).

### Antibodies, immunofluorescence, and imaging

Western blot analysis for Akt (9272, Cell Signaling Technology), phospho-Akt (9271, Cell Signaling Technology), GSK-3β (9315, Cell Signaling Technology), phospho-GSK-3β (9323, Cell Signaling Technology), and β-catenin (sc-7199, Santa Cruz Biotechnology) proteins in protein lysates from E14.5 hind limbs were performed as described (Duan et al., 2015). Antibodies to the type II collagen (Col II) triple helical domain (THD), type IIA procollagen (Col IIA) and the type IIB procollagen (pro-Col IIB) and their use in double-labeled IF to analyze the cartilage matrix are as reported (Patra et al., 2014a, 2007). IF for PECAM-1, Runx2, type I collagen (Col I), osteocalcin, Cxcl-12 and Gremlin1 proteins was performed on frozen hind limb sections after fixing tissues overnight with 4% formaldehyde (Duan et al., 2015). Antibody concentrations were as follows: antibodies to PECAM-1 (BD Biosciences), osteocalcin (ab93876, Abcam), Gremlin1 (sc-18274, Santa Cruz Biotechnology) and Cxcl-12 (ab18919, Abcam) were used at 1:50; antibodies to Runx2 (ab23981, Abcam) and Col 1 (ab21286, Abcam) were used at 1:250 and 1:100, respectively. Secondary antibodies (Life Technologies) were Alexa Fluor 594-conjugated donkey anti-rabbit (A-21207) or anti-rat (A-21209), used at 1:250. Signal amplification using a TSA kit (Thermo Fisher Scientific) was used for osteocalcin and Gremlin1 antibodies only. All antibodies were tested and validated for specific staining and showed negligible background staining. Images were captured using a 60×, 1.4 NA oil immersion objective mounted on an Eclipse E800 microscope (Nikon, Melville, NY, USA) and QImaging Retiga 2000R Fast 1394 camera and deconvolved. For deconvolution imaging, MetaMorph software (Molecular Devices) was used to control the Z-motor device (Prior Scientific, Cambridge, UK), and also to capture, deconvolve images and compile them to give a final image.

### Apoptosis assays

Detection of apoptosis on formalin-fixed tissues was performed by TUNEL assay using the *in situ* cell death detection kit (Roche) according to the manufacturer's instructions. Analysis of apoptosis in the mouse bone marrow (with or without lysis of red blood cells) was performed by labeling bone marrow cells harvested from P21 hind limbs with Andy Fluor 647-Annexin V conjugate (GeneCopoeia, Rockville, MD, USA) to detect the presence of external phosphatidylserines on the surface of cells induced by apoptosis, following the manufacturer's recommended protocol. Detection of fluorescent signals was performed on BD LSR II followed by analysis using FlowJo_V10.

### Statistical analysis

Statistical data are reported as mean±s.d. A two-tailed Student's *t*-test was used to compute values. *P*<0.05 is considered statistically significant. Number of mice per genotype (*N*) in each data set is reported in figure legends.

## Supplementary Material

Supplementary information

## Supplementary Material

First Person interview
