# Supplementary material for: Site-1 protease regulates skeletal stem cell population and osteogenic differentiation in mice
Source: Biol Open. 2018 Feb 1;7(2):bio032094. doi: 10.1242/bio.032094 (PMC5861364; doi:10.1242/bio.032094)
Supplement: Supplementary information [file biolopen-7-032094-s1.pdf]

SUPPLEMENTAL MATERIALS

SUPPLEMENTAL FIGURES

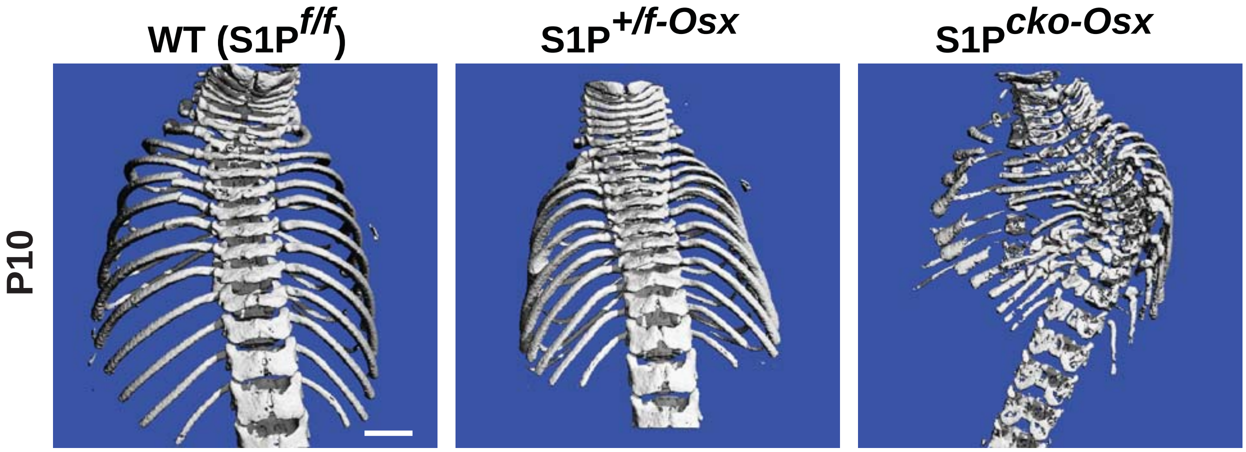

Supplemental Figure 1. Images from μCT scans of P10 WT (S1P$^{f/f}$), S1P$^{+/f\text{-}Osx}$ (Het) and S1P$^{cko\text{-}Osx}$ (Cko) mice showing scoliosis in S1P$^{cko\text{-}Osx}$. Bar: 2.5 mm.

Biology Open • Supplementary information

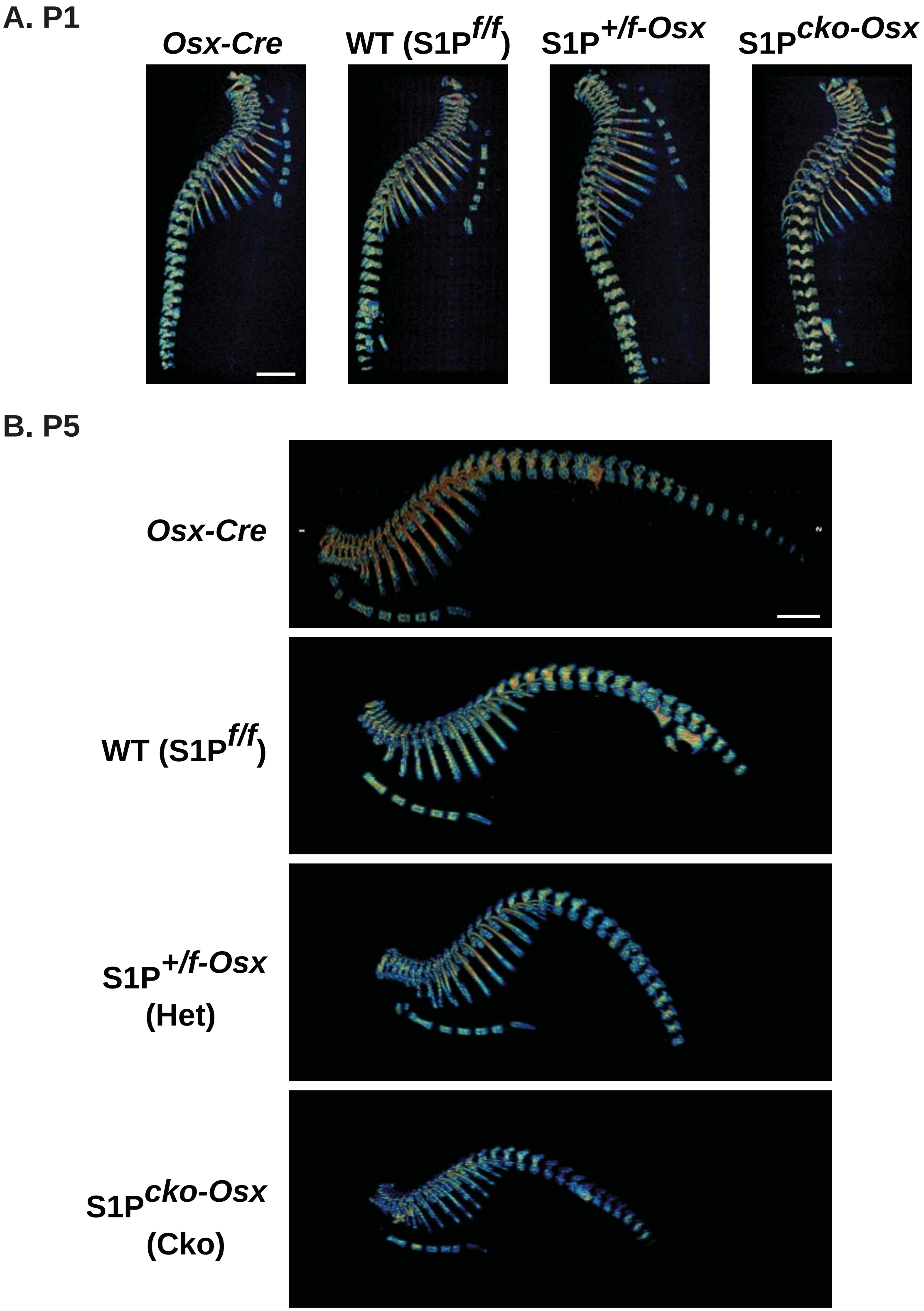

Biology Open • Supplementary information

Supplemental Figure 2. Scanned μCT images of mice axial skeletons were processed by OsiriX software and Jet color scheme using a window location of 1440 and window width of 1890 to create bone mineral density (BMD) heat maps in P1 **(A)** and P5 **(B)** *Osx-Cre*, WT ($S1P^{f/f}$), $S1P^{+/f\text{-}Osx}$ (Het) and $S1P^{cko\text{-}Osx}$ (Cko) mice. A typical representation of BMD heat maps from studies of several litters is shown. Bar (A, B): 2.5 mm.

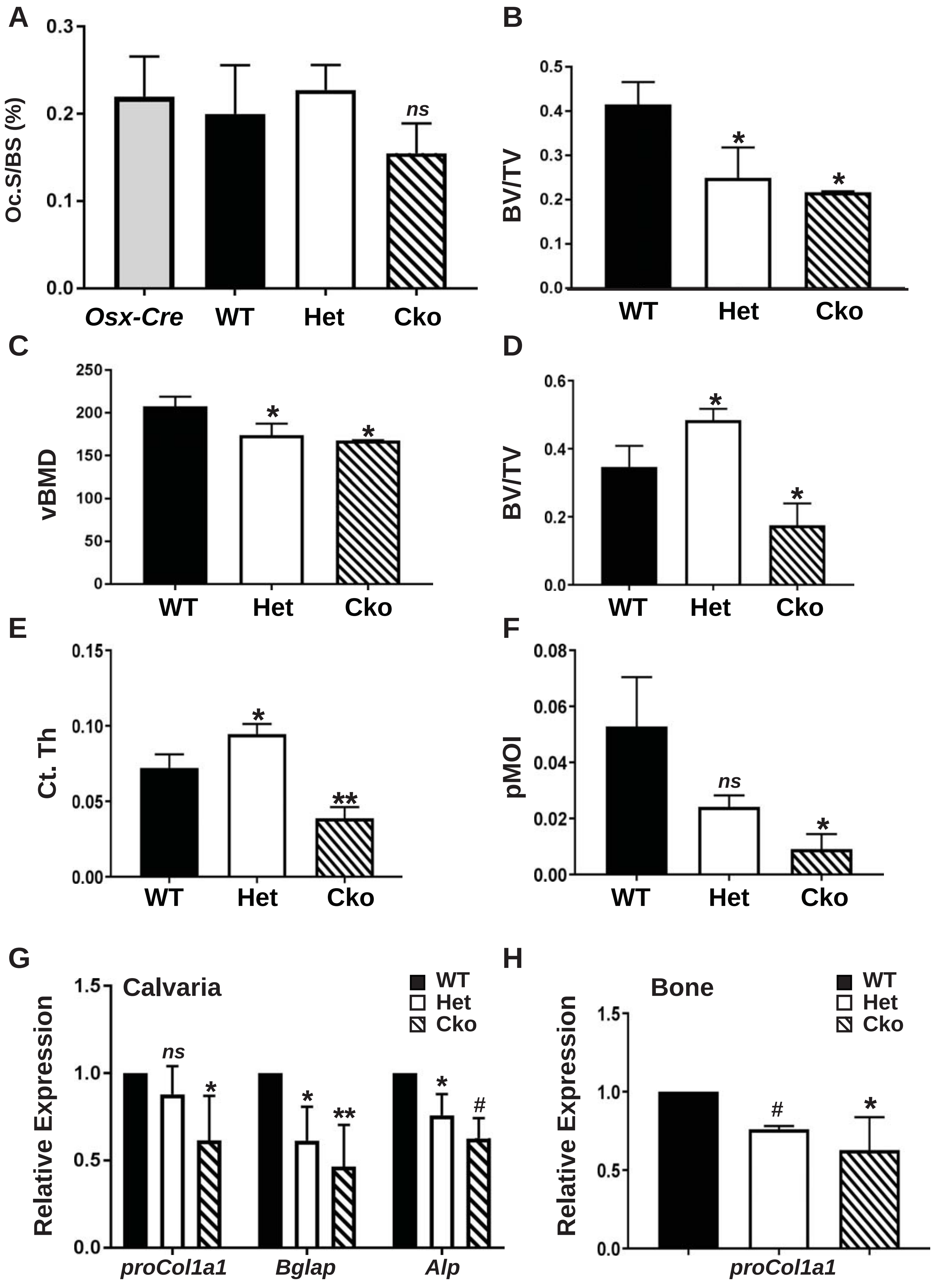

Biology Open • Supplementary information

Supplemental Figure 3. **(A)** Dynamic histomorphometric measurement of osteoclast surface/bone surface (Oc.S/BS) (mean ± s.d.; N=5) for P21 *Osx-Cre*, WT, Het and Cko mice (P = *ns* (not significant)). Morphological analysis from µCT scans for the metaphyseal trabecular bone in the distal femora **(B, C)** and mid-diaphyseal cortical bone **(D-F)** in P7 mice (mean ± s.d.; N=3; $^{*}P < 0.05$, $^{**}P < 0.01$, or *ns* when compared to WT). (BV: bone volume; TV: tissue volume; vBMD: volumetric bone mineral density in milligrams of hydroxyapatite per cubic centimeter; Ct. Th: cortical thickness; pMOI: polar moment of inertia as a measure of resistance to torsional force). Q-PCR analysis for *proCol1a1*, *Bglap*, and *Alp* with RNA harvested from P21 calvaria **(G)** and long bones (femur/tibia with bone marrow flushed out) **(H)** (mean ± s.d.; N=3, $^{*}P < 0.05$, $^{**}P < 0.01$, $^{\#}P < 0.005$ or *ns*, when compared to WT).

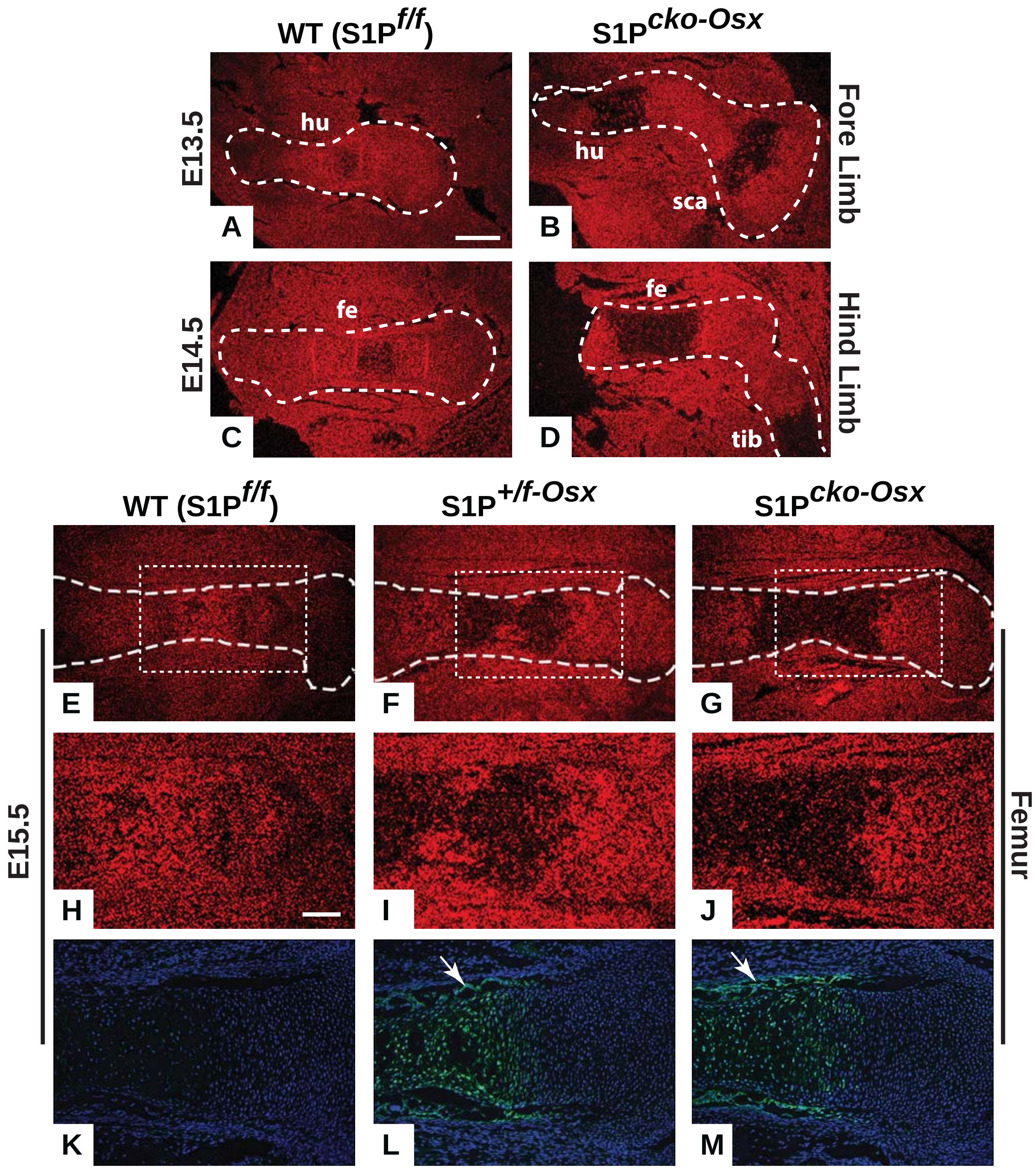

Biology Open • Supplementary information

Supplemental Figure 4. (A-G) *In situ* hybridization analysis for *Mbtps1-exon 2* at E13.5 (A and B, in fore limb) and E14.5 (C and D, in hind limb) in WT and S1P$^{cko\text{-}Osx}$ mice, and at E15.5 in WT, S1P$^{+/f\text{-}Osx}$ and S1P$^{cko\text{-}Osx}$ mice (E-G, femur). Signals from *Mbtps1-exon 2* are shown in red. Notice the loss of expression in the pre-hypertrophic/hypertrophic zones in the growth plate (hu: humerus; sca: scapula; fe: femur; tib: tibia). Panels **H-J** are higher magnification images of panels **E-G** of the re ion demarcated by the dashed rectangle and are shown in the same magnification as panels **K-M**. **(K-M)** GFP expression (due to expression of the GFP-Cre fusion protein from the *Sp7* (*Osx*) promoter) in E15.5 femora in S1P$^{+/f\text{-}Osx}$ (Het) and S1P$^{cko\text{-}Osx}$ (Cko) mice, absent in WT, showing the tissue distribution of *Osx-Cre* activity and its overlap with *Mbtps1-exon2* ablation **(E-J)**. Arrow points to GFP expression in the perichondrium indicating *Mbtps1-exon 2* deletion in perichondrial bone progenitors. Bar (A-G): 250 µm; (H-M): 100 µm.

Biology Open • Supplementary information

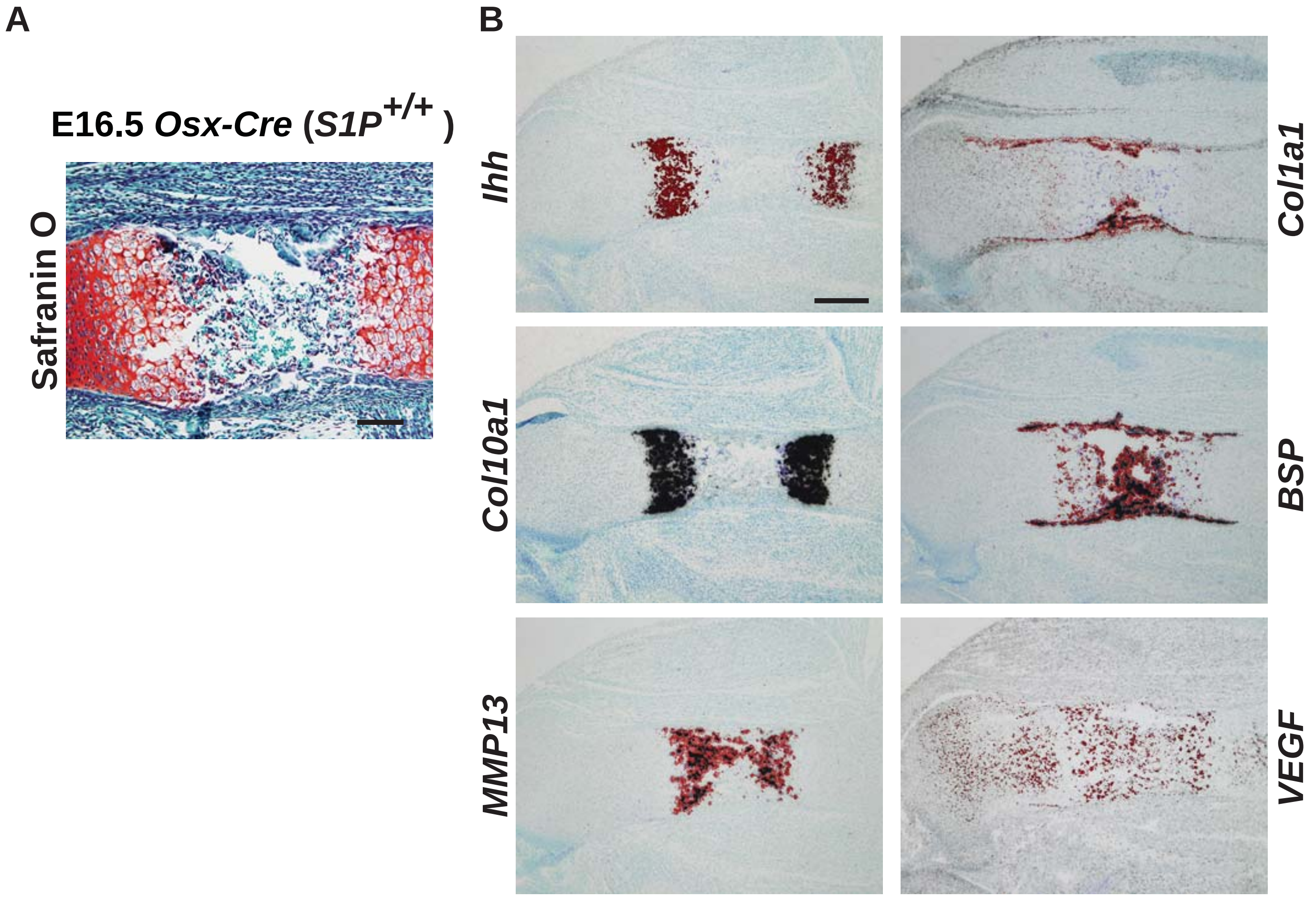

Supplemental Figure 5. **(A)** Safranin O, Fast green, and hematoxylin stained femur from a control *Osx-Cre* (*S1P*$^{+/+}$) mouse at E16.5. Bar: 100 µm. **(B)** *In situ* hybridization analyses in *Osx-Cre* control mouse in E16.5 femur for *Ihh*, *Col10a1*, *MMP13*, *Cola1*, *BSP*, and *VEGF*. Bar (all panels): 250 µm.

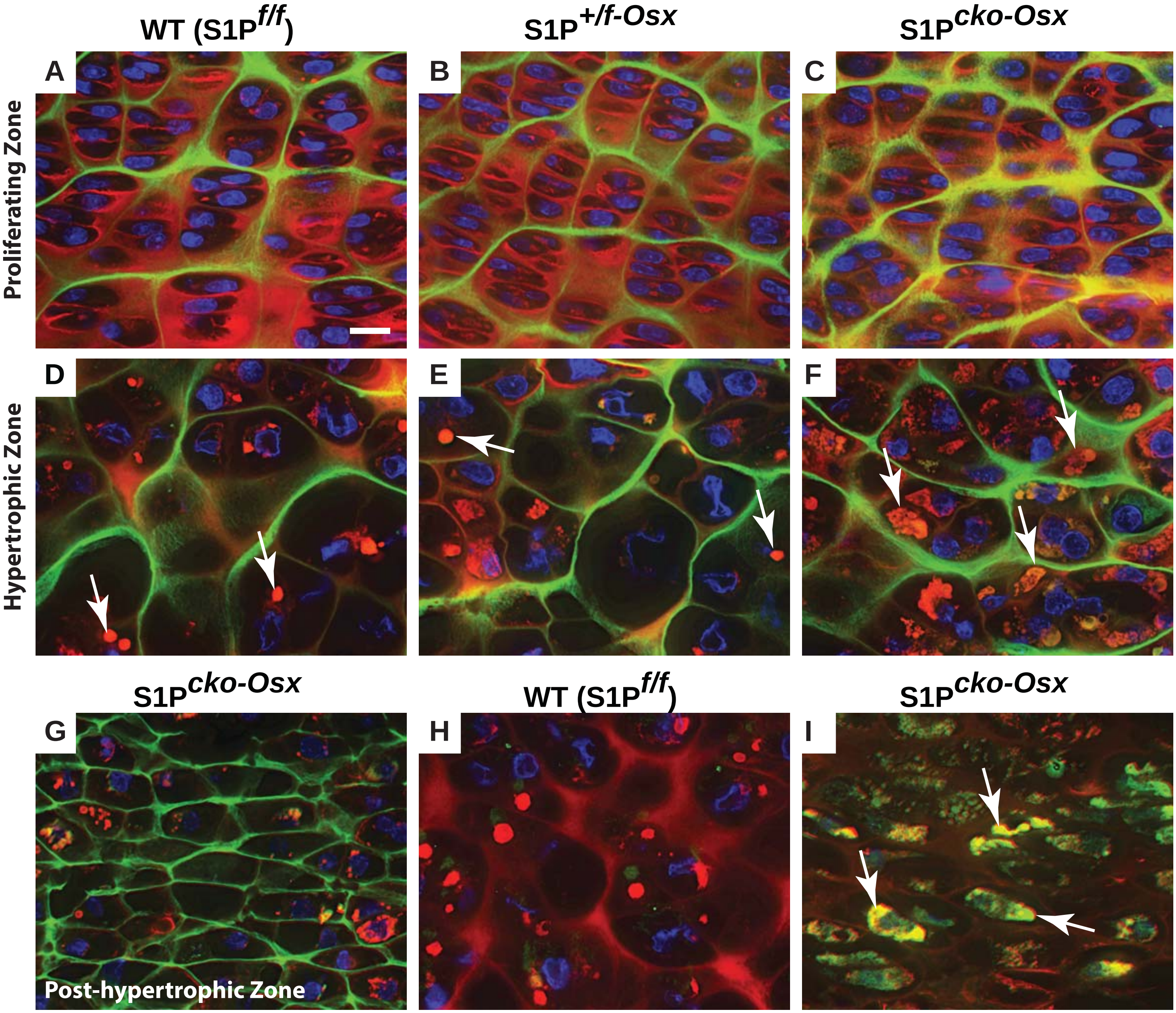

Biology Open • Supplementary information

Supplemental Figure 6. Entrapment of pro-Col IIB in the pre-hypertrophic/hypertrophic chondrocytes in S1P$^{cko\text{-}Osx}$. (**A-G**) Double-labeled immunofluorescence for Col II THD (red) and Col IIA (green) in E16.5 femora in WT **(A, D)**, S1P$^{+/f\text{-}Osx}$ (**B, E**) and S1P$^{cko\text{-}Osx}$ **(C, F)** are shown for proliferating cells (**A-C**), pre-hypertrophic/hypertrophic chondrocytes (**D-F**), and the post-hypertrophic zone (**G**, S1P$^{cko\text{-}Osx}$ only). Arrows point to Col II THD clumps in WT **(D)** and S1P$^{+/f\text{-}Osx}$ **(E)** hypertrophic matrix, or the intracellularly trapped Col II in S1P$^{cko\text{-}Osx}$ **(F)**. **(H, I)** Double-labeled immunofluorescence for pro-Col IIB (green) and Col II THD (red) in E16.5 femora in WT (**H**) and S1P$^{cko\text{-}Osx}$ (**I**). Arrows (**I**) point to yellow colocalization signals from pro-Col IIB and THD indicating pro-Col IIB entrapment. Bar (all panels): 10 μm.

Biology Open • Supplementary information

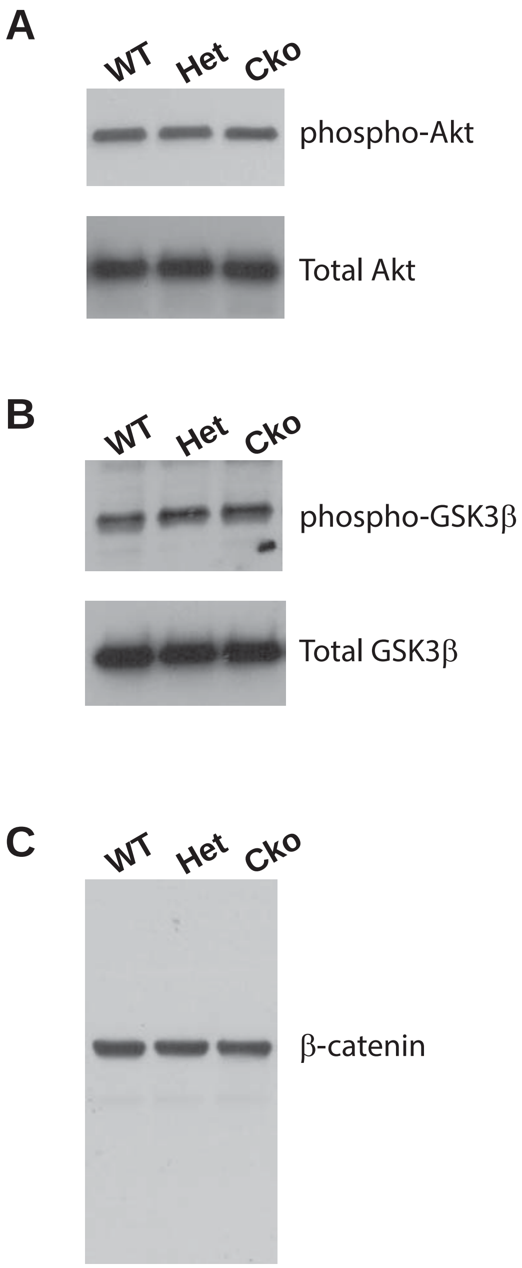

Supplemental Figure 7. Immunoblot analysis of Wnt/β-catenin signaling components in embryonic bone. E14.5 forelimbs were harvested from WT, S1P$^{+/f\text{-}Osx}$ (Het) and S1P$^{cko\text{-}Osx}$ (Cko) mice, proteins extracted and equal amounts of limb lysates loaded and analyzed by western blot with the antibodies indicated.

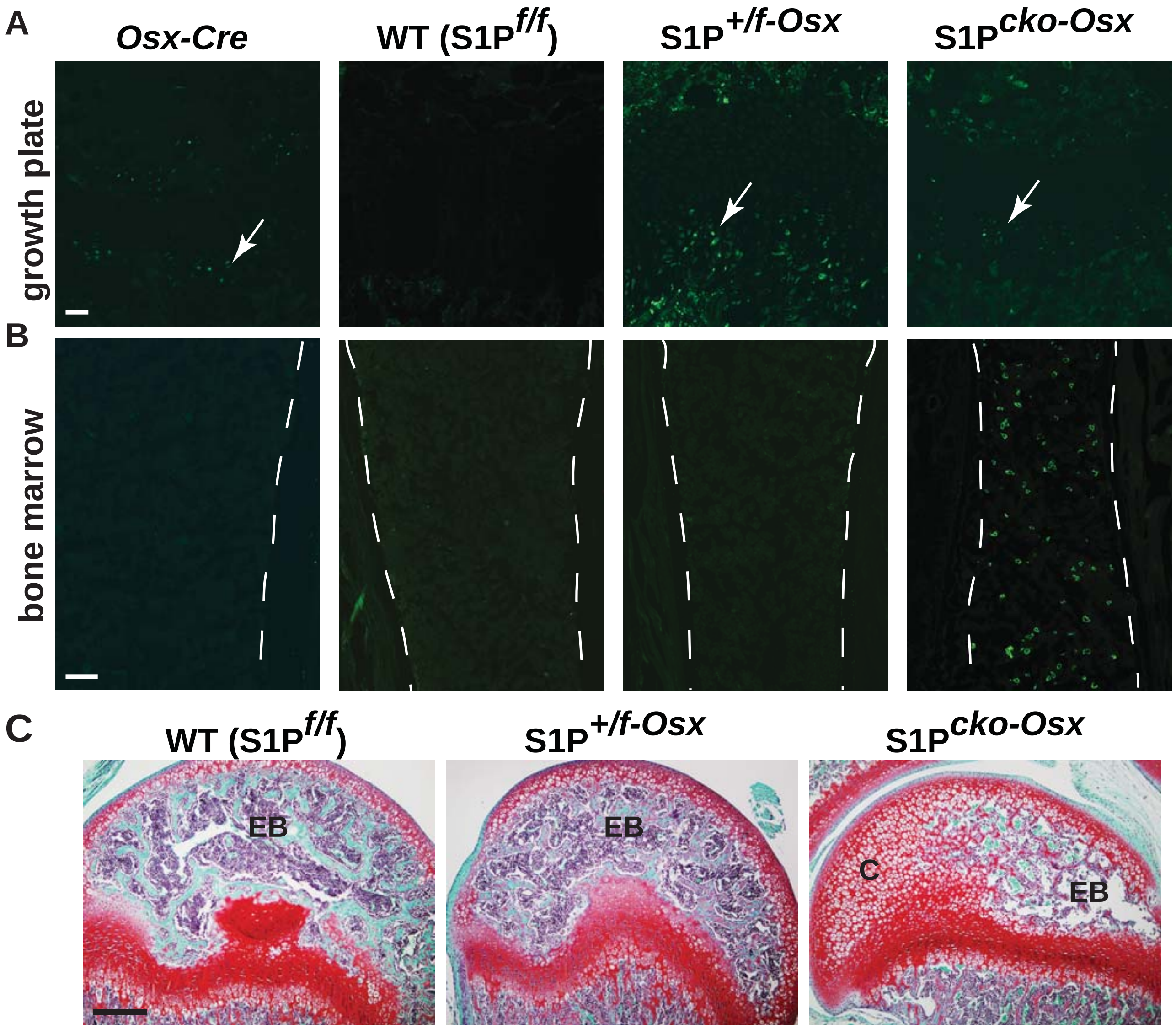

Supplemental Figure 8. Distribution of GFP^{+} cells in the growth plate **(A)** and bone marrow **(B)** in P21 *Osx-Cre*, WT (S1P$^{f/f}$), S1P$^{+/f\text{-}Osx}$ (Het) and S1P$^{cko\text{-}Osx}$ (Cko) mice. Dashed lines show the junction of bone marrow with cortical bone. Arrows point to the loc tion of GFP^{+} cells in the pre-hypertrophic/hypertrophic zone of the growth plates. **(C)** Safranin O stained sections from P21 mice showing the secondary ossification center in the proximal humerus. Notice the smaller size of the humeral head in S1P-ablated mice and the incomplete removal of cartilage in S1P$^{cko\text{-}Osx}$ (C: cartilage; EB: epiphyseal bone). Bar (A): 50 μm; (B): 100 μm; (C): 250 μm.

Biology Open • Supplementary information
