# Supplementary material for: Site-1 protease regulates skeletal stem cell population and osteogenic differentiation in mice
Source: Biol Open. 2018 Feb 1;7(2):bio032094. doi: 10.1242/bio.032094 (PMC5861364; doi:10.1242/bio.032094)
Supplement: First Person interview [file biolopen-7-032094-s2.pdf]

FIRST PERSON

First person – Debabrata Patra

First Person is a series of interviews with the first authors of a selection of papers published in Biology Open, helping early-career researchers promote themselves alongside their papers. Debabrata Patra is first author on 'Site-1 protease regulates skeletal stem cell population and osteogenic differentiation in mice', published in BiO. Debabrata is an Associate Professor in the Department of Orthopaedic Surgery at Washington University School of Medicine, USA, conducting molecular analysis of skeletal development and associated pathologies.

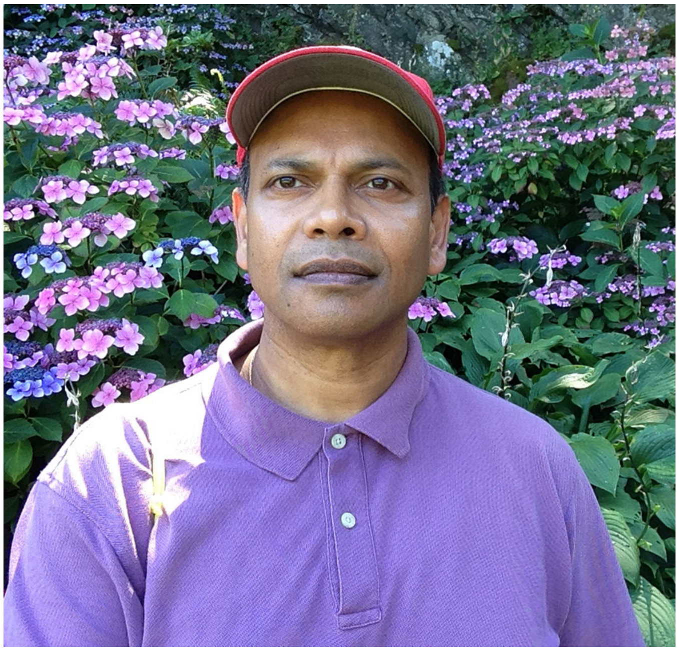

Debabrata Patra

What is your scientific background and the general focus of your lab?

With a mostly academic training (though I have had a stint in industry as well) one ends up acquiring experience in more than one sub-field under the broad umbrella of biological sciences. In my student and postdoctoral years I was mostly a molecular biologist/biochemist by training. But of late I would describe myself as a developmental and cell biologist who is also using a number of other fascinating technological developments, such as next-generation sequencing and imaging, to achieve a molecular understanding of skeletal biology and its related pathologies. I believe that this will help develop a foundation on which rational therapies may be designed to treat skeletal diseases, including attempting regenerative measures. Skeletal development is a very complex developmental process and fortunately for me we have a number of knockout mouse models that cover some of the primary features of the mammalian skeleton, namely, cartilage, bone and spine. What is central to these mouse models is the loss of site-1 protease in the skeletal elements. Thus our mutant mouse models can be studied in depth to unravel some of the molecular mysteries of skeletal development.

How would you explain the main findings of your paper to non-scientific family and friends?

We have discovered that the absence of a protein, called site-1 protease (S1P) in bone progenitor cells in the mouse skeleton prevents the formation of strong bones in these mice. These bones are fragile, break easily and are also smaller in size, resulting in short-statured mice. In quantitating a number of parameters we found that in the absence of S1P, bone progenitor cells have difficulty differentiating or maturing into osteoblasts. Osteoblasts are cells that make bones in our bodies. This means that S1P regulates bone formation at a very crucial fundamental level. Currently we do not know the molecular pathways that S1P controls during osteoblast formation and what other proteins it crosses path with in performing its functions. Ongoing experiments in the laboratory are investigating these aspects.

"I think that people forget that no matter how sound a hypothesis may sound on paper, only a well-planned experiment at the bench will inform you of the truth."

What are the potential implications of these results for your field of research?

At the most basic level we are trying to understand skeletal development. We hope that we can harness this knowledge to treat not only those rare skeletal diseases but even common ones such as osteoarthritis (OA, damage of articular joint cartilage), osteoporosis (bone loss) and scoliosis (curving of spine), which are so common in the aging public. We are hoping that one can use this knowledge to cure or delay the onset of these diseases, or allow to grow parts such as a functional cartilage outside the body, which can be used as replacement parts in humans with terminal OA. As skeletal biologists, we also hope that the designing of disease-modifying drugs, which are absent for OA, may find its genesis in this field of research.

What has surprised you the most while conducting your research?

In general, what surprises me the most is how often the results of a carefully planned experiment belie our expectations. In my career I can often recall how I was told not to expect a particular result or not to follow a particular research direction as it would not reveal anything new, and how often this was not true for me; in hindsight I was so glad that I did not listen. I think that

Debabrata Patra's contact details: Department of Orthopaedic Surgery, Washington University School of Medicine, St Louis, USA.

E-mail: debabratapatra@wustl.edu

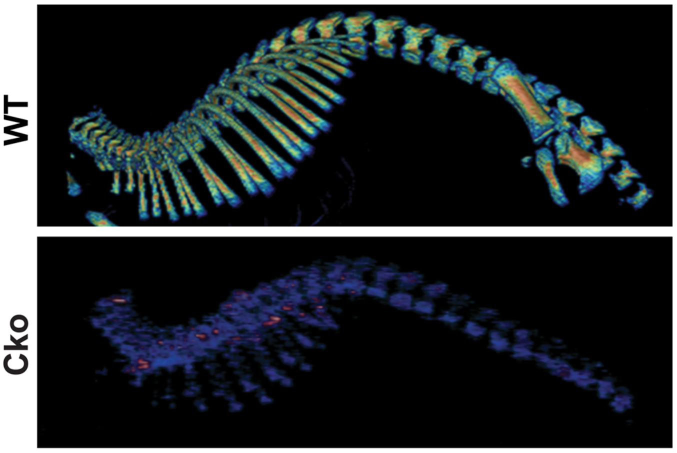

Loss of site-1 protease in the osterix lineage in mice (Cko) results in significant bone loss when compared to wild type (WT). Shown here are bone mineral density (BMD) heat maps where yellowish-orange indicates high, and blue low, values for BMD.

people forget that no matter how sound a hypothesis may sound on paper, only a well-planned experiment at the bench will inform you of the truth.

What, in your opinion, are some of the greatest achievements in your field and how has this influenced your research?

That one can attempt to grow an organ or tissue outside the body is fascinating to me, definitely utopian and magical. It's like you have unravelled one of nature's long-held mysteries that nature did not want to give up easily. A number of laboratories are attempting to grow cartilage in the laboratory with the hope that these will be implanted on patients with terminal OA. I have attended some of their talks where people have described their research. Listening to their achievements and successes and how close they are coming every day to actually performing a successful cartilage implant in humans to treat OA is definitely encouraging. The other aspect is the advent of stem cells in the field of orthopaedics. Scientists have discovered stem cells and their profound impact on development and organogenesis, but yet little is known about how they are regulated and controlled in the various differentiation programs towards bone or cartilage. My current work published in BiO shows the intersection of S1P with skeletal stem cells and this has increased my curiosity about stem cells and what might S1P be doing in this context.

What changes do you think could improve the professional lives of early-career scientists?

One change is the mindset of young scientists. Most of us because of our academic background think that following a research career is the only path we are trained for. But primarily what we are trained to do is to think objectively through a problem, design solutions to alleviate these issues, and develop good communication skills, both oral and written. These are valuable skill sets that can be exploited in non-research track as well and most of us do not explore them as a viable option for the next step in our career should it become difficult to establish oneself in a pure research discipline.

What's next for you?

I am trying to identify a potential mechanistic pathway where S1P is involved in bone development. Simply put, I am on the hunt for identifying a substrate for S1P. S1P is a proprotein convertase, a protease that processes immature proteins to mature proteins. This would allow us to understand S1P's contribution to bone development, which again I hope can be harnessed to treat bone disorders such as osteoporosis.

Reference

Patra, D., DeLassus, E., Mueller, J., Abou-Ezzi, G. and Sandell, L. J. (2018). Site-1 protease regulates skeletal stem cell population and osteogenic differentiation in mice. *Biol. Open* **7**, doi:10.1242/bio.032094.

Biology Open
